# Amnion-derived hydrogels as a versatile platform for regenerative therapy: from lab to market

**DOI:** 10.3389/fbioe.2024.1358977

**Published:** 2024-02-26

**Authors:** Golara Kafili, Hassan Niknejad, Elnaz Tamjid, Abdolreza Simchi

**Affiliations:** ^1^ Center for Nanoscience and Nanotechnology, Institute for Convergence Science and Technology, Sharif University of Technology, Tehran, Iran; ^2^ Department of Pharmacology, School of Medicine, Shahid Beheshti University of Medical Sciences, Tehran, Iran; ^3^ Department of Nanobiotechnology, Faculty of Biological Sciences, Tarbiat Modares University, Tehran, Iran; ^4^ Department of Materials Science and Engineering, Sharif University of Technology, Tehran, Iran; ^5^ Center for Bioscience and Technology, Institute for Convergence Science and Technology, Sharif University of Technology, Tehran, Iran

**Keywords:** human-derived materials, decellularized extracellular matrix, injectable hydrogels, 3D bioprinting, nanomedicine, safety

## Abstract

In recent years, the amnion (AM) has emerged as a versatile tool for stimulating tissue regeneration and has been of immense interest for clinical applications. AM is an abundant and cost-effective tissue source that does not face strict ethical issues for biomedical applications. The outstanding biological attributes of AM, including side-dependent angiogenesis, low immunogenicity, anti-inflammatory, anti-fibrotic, and antibacterial properties facilitate its usage for tissue engineering and regenerative medicine. However, the clinical usage of thin AM sheets is accompanied by some limitations, such as handling without folding or tearing and the necessity for sutures to keep the material over the wound, which requires additional considerations. Therefore, processing the decellularized AM (dAM) tissue into a temperature-sensitive hydrogel has expanded its processability and applicability as an injectable hydrogel for minimally invasive therapies and a source of bioink for the fabrication of biomimetic tissue constructs by recapitulating desired biochemical cues or pre-defined architectural design. This article reviews the multi-functionality of dAM hydrogels for various biomedical applications, including skin repair, heart treatment, cartilage regeneration, endometrium regeneration, vascular graft, dental pulp regeneration, and cell culture/carrier platform. Not only recent and cutting-edge research is reviewed but also available commercial products are introduced and their main features and shortcomings are elaborated. Besides the great potential of AM-derived hydrogels for regenerative therapy, intensive interdisciplinary studies are still required to modify their mechanical and biological properties in order to broaden their therapeutic benefits and biomedical applications. Employing additive manufacturing techniques (e.g., bioprinting), nanotechnology approaches (e.g., inclusion of various bioactive nanoparticles), and biochemical alterations (e.g., modification of dAM matrix with photo-sensitive molecules) are of particular interest. This review article aims to discuss the current function of dAM hydrogels for the repair of target tissues and identifies innovative methods for broadening their potential applications for nanomedicine and healthcare.

## Graphical Abstract

**Figure:**
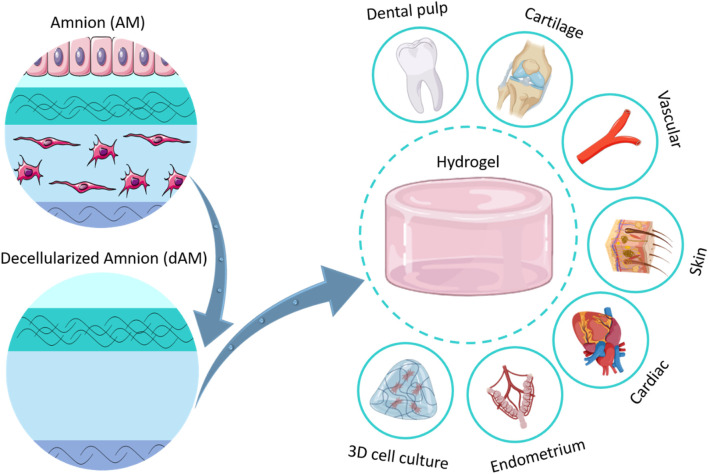
The Figure was partly generated using Servier Medical Art, provided by Servier, licensed under a Creative Commons Attribution 3.0 unported license.

## 1 Introduction

Damage to tissues can be caused due to several reasons, such as sudden or unexpected accidents, genetic disorders, congenital anomalies, and trauma (Krafts, 2010). In simple cases, the body can recover tissue through inflammation, cell proliferation, and tissue regeneration (Poss, 2010). However, in serious injuries, medical interventions such as transplanting tissue substitutes (i.e., autografts, allografts, or xenografts) or stimulating tissue regeneration using tissue-engineered constructs (TECs) are required (Gaharwar et al., 2020). Among various TECs or bio-derived scaffolds, the amniotic membrane also named as amnion (AM) has gained great attention for tissue engineering (TE) and regenerative medicine (RM), thanks to its exquisite biological characteristics, including excellent biocompatibility, anti-bacterial, anti-inflammatory, anti-fibrotic, immunomodulatory and angiogenic properties (Munoz-Torres et al., 2023). AM meets all basic requirements of TE, including a rich source of stem cells, growth factors, and bioactive molecules as well as extracellular matrix (ECM) components that further highlight its eligibility for therapeutic purposes (Jahanafrooz et al., 2023).

AM has been discovered as a potential candidate for skin regeneration and reconstruction surgeries since the early 20th century (John, 2003). The first report of using AM as a skin replacement was done by Davis in 1910 (Davis, 1910). Later in 1913, AM was used by Stern et al. for treating burn and ulcerated wounds (Stern, 1913). Since then, AM has shown promising results in the repair of various tissues including, the heart, ocular surface, vascular, cartilage, uterus, etc (Cornwell et al., 2009; Horn et al., 2019; Hossain et al., 2020; Lacorzana, 2020). Despite the advantageous clinical results obtained by grafting AM, there are some challenges for surgeons to handle thin AM tissue without tearing or folding, which limits the incorporation of fresh AM into routine clinical applications to some extent (Murphy et al., 2017; Dadkhah Tehrani et al., 2021). Besides, the dense structure of the AM tissue is known to limit the penetration of cells to the wounded site (Nasiry et al., 2021; Nasiry et al., 2022; Khalatbary et al., 2023; Nasiry et al., 2023). One of the methods to solve these issues was to use its dehydrated form, which is easier to handle and can be stored at room temperature with a shelf life of about 5 years (Fetterolf and Snyder, 2012). Although the dehydrated AM tissue has shown promising outcomes for treating wounds, it lacks efficacy for the treatment of large or irregularly shaped injuries (Murphy et al., 2017). Therefore, processing of the AM tissue into a hydrogel can afford easier handling by gelation inside any nonuniform defect injuries. To this end, some researchers have developed a gel formula based on the incorporation of AM powder or AM extracts into a hydrogel solution (Murphy et al., 2017; Rahman et al., 2019; Islam et al., 2023). In other studies, the development of an injectable hydrogel through the enzymatic digestion of decellularized amnion (dAM) powder in a mildly acidic solution has been performed (Ryzhuk et al., 2018). This category of hydrogels forms physical crosslinking by incubation of the pH-neutralized dAM solution at the physiologic temperature (Li et al., 2022).

The applications of AM tissue and AM-derived stem cells in different fields of TE and RM are well-covered in recent literature (Jafari et al., 2021; Elkhenany et al., 2022; Hu et al., 2023). This review aims to comprehensively explain the advanced multifaceted therapeutic functions of hydrogels derived from AM or dAM for tissue regeneration. The structural features and components of AM are introduced and various biological functions of the tissue are presented and discussed. The commercial products based on AM, including their features and shortcomings, are also demonstrated. Besides, the most relevant fabrication processes of the AM/dAM hydrogels and their applications in the engineering and regeneration of heart, skin, cartilage, vascular, endometrium and fetal membrane, and dental pulp are critically reviewed. Finally, recent insights into the modification of AM/dAM-derived hydrogels, such as designing hybrid hydrogels by incorporating supporting polymeric constituents, rapid photocrosslinking process through grafting light-sensitive functional groups, and nanoparticles, are concluded.

## 2 Structural and biochemical features of amnion

**FIGURE 1:**
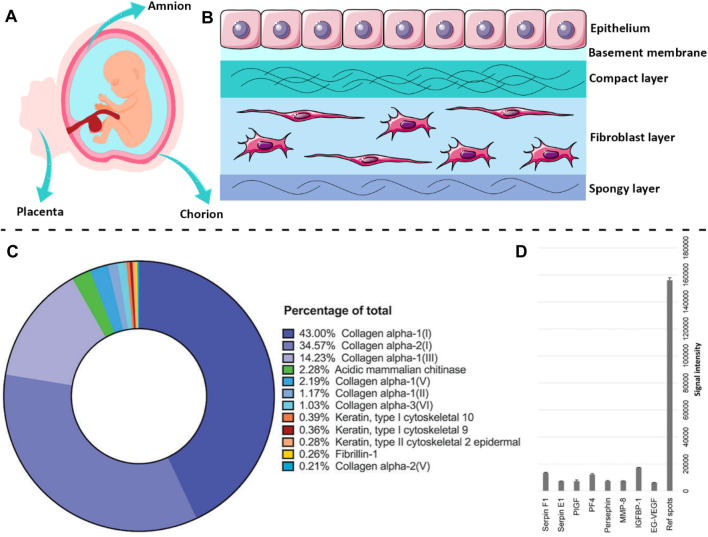
The structural architecture of AM tissue and biochemical components within the dAM matrix. A scheme of human **(A)** placenta and **(B)** AM membrane. **(C)** The main proteins of the dAM matrix evaluated by mass spectroscopy [Reprinted from Comperat et al. (2023), Copyright (2023), with permission from Wiley-VCH GmbH]. **(D)** The most frequent cytokines in dAM hydrogel assessed by the angiogenesis array [Reprinted from Ryzhuk et al. (2018), Copyright (2023), with permission from Elsevier]. Panel 1B was partly generated using Servier Medical Art, provided by Servier, licensed under a Creative Commons Attribution 3.0 unported license.

The human placenta is considered a biowaste for hospitals and is discarded after the baby is born (Mamede et al., 2012). Hence, the placenta is an available and cost-effective source of tissue that does not require extreme ethical considerations for its clinical usage (Haghshenas et al., 2022). AM is the innermost fetal membrane of the placenta that protects the fetus during pregnancy (Figure 1A). The outer layer is called the chorion plate, which contacts with the mother’s cells (Salah et al., 2020). AM is a thin (0.02–0.5 mm) and translucent tissue that does not possess any muscles, nerves, or vessels, and its nutrition occurs due to diffusion (Toda et al., 2007). As depicted in Figure 1B, the human AM is composed of three main layers, including the epithelium, basement membrane, and stromal (Niknejad et al., 2008). The epithelial cells secrete a wide range of growth factors (GF) and cytokines, including epidermal GF (EGF), vascular endothelial GF (VEGF), keratinocyte GF (KGF), basic fibroblast GF (bFGF), alpha- and beta-transforming GF (TGF-α, TGF-β), interleukin-8 (IL-8), angiogenin, serine protease inhibitor (serpin) E1, insulin-like GF (IGF), and their binding proteins (IGFBP) (Favaron et al., 2015). These cells are firmly attached to the basement membrane that is a supply of sulfated proteoglycans (e.g., heparan sulfate) and as a permeable barrier allows the transport of several macromolecules, including α-actinin, spectrin, vimentin, laminin, desmoplakin, cytokeratins, etc. (Ohno et al., 1983; Liu et al., 2019; Shariatzadeh et al., 2021). The basement membrane of AM also contains collagen (type III, IV, and V) and various non-collagenous glycoproteins, such as elastin, laminin, nidogen, fibronectin, and vitronectin as well as glycosaminoglycans like hyaluronic acid (Arki et al., 2023; Fitriani et al., 2023). The stromal layer is composed of three sublayers, including 1) compact layer, 2) fibroblast layer, and 3) intermediate layer (Dadkhah Tehrani et al., 2021). The compact layer which is located beneath the basement membrane is a cell-free connective tissue that mainly consists of collagen (type I, III, V, and VI) (Sadler, 2022). The next layer, which is the thickest layer of AM, contains mesenchymal fibroblast-like cells and collagen-rich ECM which significantly affects the tensile strength of the AM tissue (Mamede et al., 2012). The outermost layer is a cell-free nonfibrillar matrix containing type III collagen, proteoglycans, glycoproteins, and hydrated glycoproteins that separate the AM from the underlying chorion (Toda et al., 2007). This layer is called the “spongy layer” because the proteoglycans and glycoproteins components inside this layer produce a spongy appearance (Fénelon et al., 2021). It is noteworthy to mention that collagen I and II and elastin regulate the tensile strength of the AM, while collagen III is responsible for the elasticity (Mamede et al., 2012). Therefore, it can be concluded that AM is a rich source of various proteins, GF, and GAGs, including collagen (types I, III, IV, V, and VI), fibronectin, elastin, nidogen, and hyaluronic acid that support the proliferation and differentiation of cells, and encourage re-epithelialization (Rana et al., 2020).

Decellularization of native tissues and organs is a prerequisite step for their safe implementation for TE applications, since the resident cells may cause intense host immunologic reactions after transplantation and transplant rejection (Bhattacharjee et al., 2020). Accordingly, various mechanical, chemical, and enzymatic techniques and a combination of these methods have been adopted for the successful removal of cells from tissues (Arrizabalaga and Nollert, 2018). Although the decellularization process is quite a promising tool for diminishing cellular components of tissues and organs, it may also cause the loss of some bioactive ECM components (Kim et al., 2020). Hence, it is important to choose a decellularization agent in a way that provides sufficient cell removal without seriously damaging the ECM structure and existing biochemical cues (Kim et al., 2017). Regardless of losing some of the ECM components during the decellularization process, a sufficient amount of bioactive components still remain within the dAM matrix to provide reliable functionality for TE applications (Wassmer and Berishvili, 2020). As reported by Comperat et al. (2023), the dAM matrix is mainly composed of structural proteins such as collagen, and fibrillin-1, and cytoskeletal-associated proteins (e.g., keratin-type I) (Figure 1C). The results of the angiogenesis assay also suggest that the dAM matrix is a valuable source of various proteins and GF, including placenta (PlGF), platelet factor 4 (PF4), insulin-like binding protein 1 (IGFBP-1), and endocrine gland-derived vascular endothelial (EG-VEGF) (Figure 1D).

## 3 Bio-functionality of amnion

**FIGURE 2:**
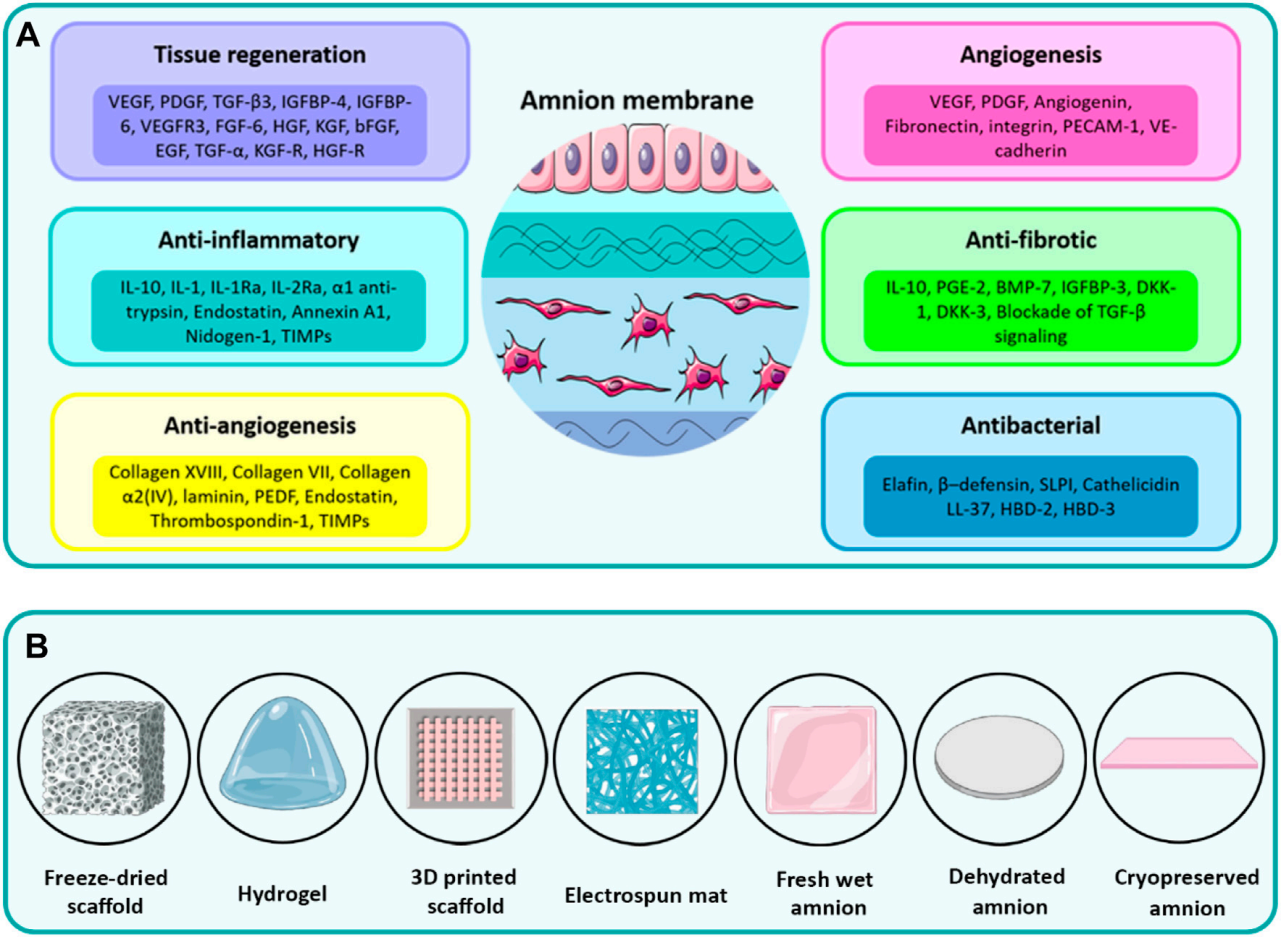
**(A)** The diverse therapeutic properties of the amnion induced by different inherent cytokines, growth factors, and ECM components. **(B)** Various types of processing methods to fabricate biomedical scaffolds from AM. Panel 2A was partly generated using Servier Medical Art, provided by Servier, licensed under a Creative Commons Attribution 3.0 unported license.

Although AM is an avascular tissue, it plays an important role as a tissue for the production of several bioactive molecules, including growth factors, cytokines, and vasoactive peptides (Cunningham et al., 2014). As schematically shown in Figure 2A, AM is a biocompatible tissue that provides outstanding biological characteristics, such as low immunogenicity, anti-inflammatory, anti-fibrotic, and anti-bacterial properties along with side-dependent angiogenesis (Elkhenany et al., 2022). In several studies, for example, Favaron et al. (2015), Babajani et al. (2022a), Babajani et al. (2022b), Biniazan et al. (2022), Jafari et al. (2023), AM-derived stem cells with angiogenic, immunosuppressive, and anti-tumoral properties have been found of interest for tissue regeneration. The various types of ECM components are mostly secreted from resident cells of AM to form different sublayers of AM as described thoroughly in the previous section. In the following, the regenerative effects induced by bioactive constituents of the amnion matrix, e.g., proteins, growth factors, and cytokines, are presented and discussed. It is noteworthy that the biological properties of AM tissue, as similar to other native tissues, may be more pronounced than the decellularized AM (dAM) because the decellularization process is potentially susceptible to the loss of some part of the bioactive and functional constituents of native tissues (Elkhenany et al., 2022). However, exploiting an optimized decellularization process may guarantee the preservation of a considerable portion of these biochemical cues within dECM biomaterials (Saldin et al., 2017).

### 3.1 Anti-inflammatory

So far, different mechanisms have been proposed for explaining the anti-inflammatory effect of AM by different generations of researchers in this field. The anti-inflammatory of AM is ascribed to its inhibiting effect on the infiltration of inflammatory cells to an injured site (Shimmura et al., 2001). The soluble factors secreted from epithelial cells (e.g., interleukin-1 receptor antagonist (IL-1Ra), IL-2Ra, IL-10, and endostatin) are also effective (Hao et al., 2000; Li et al., 2005). These factors hinder the immune cells and reduce angiogenesis and tumor growth by restricting the proliferation of endothelial cells. Particularly, IL-10 suppresses the activity of pro-inflammatory cytokines, such as IL-1, IL-6, IL-8, interferon-γ (IFN-γ), and tumor necrosis factor-alpha (TNF-α) (Silini et al., 2017; Koelink et al., 2020). The anti-inflammatory property of AM is further speculated to be due to the suppression effect of its stromal matrix on the expression of IL-1α and IL-1β pro-inflammatory cytokines (Niknejad et al., 2008). Amnion also enables the expression of tissue inhibitors of metalloproteinase (TIMPs)-1, 2, 3, and 4, which can suppress the ECM digestive function of matrix metalloproteinases (MMPs) produced by macrophages and polymorphonuclear cells (Kim et al., 2000). The ability of AM to combat inflammation can also be explained by its protease inhibitors, such as α1 anti-trypsin and inter-α-trypsin (Elkhenany et al., 2022). Antimicrobial peptides (AMPs) within AM, such as human beta-defensins (HBDs), cathelicidin, and histones are capable of attenuating inflammation induced by lipopolysaccharide (LPS) (Guaní-Guerra et al., 2010). Furthermore, the protease and elastase inhibitory effects of elafin and secretory leucocyte proteinase inhibitor (SLPI) control the inflammatory responses in the surfaces of tissues suspected of contamination (Zare-Bidaki et al., 2017).

### 3.2 Antibacterial and anti-viral activity

AM exhibits promising results in controlling the infection of wounds because it not only acts as an adherent shield over an injured tissue to inhibit the infiltration of bacteria but also regulates the expression of molecules with antibacterial or antiviral properties (King et al., 2007). Ni et al. (1997) have stated that AM offers anti-viral properties due to the presence of cystatin E in its matrix which is an analog for cysteine proteinase inhibitor. The collagen fibers within the AM matrix can also contribute to reduced bacterial accumulation and inhibited hematoma formation, owing to their hemostatic property (Baradaran Rafii et al., 2007). The epithelial cells of AM can secrete AMPs, such as β-defensin 1-3 (HBD 1–3), LL37, histone H2B, and elafin which are helpful in the management of wound infection (Kim et al., 2002; Tehrani et al., 2013). The elastase inhibitors expressed by AM, including elafin and SLPI proteins that contain Whey acidic peptide (WAP) motifs, are also responsible for anti-inflammatory and antibacterial properties (Mohan et al., 2017; Tehrani et al., 2017; Lohajaroensub et al., 2022). Moreover, the AM contains various AMPs, including human neutrophil peptides 1-3, calprotectin (MRP8/14), lysozyme, and ubiquitin that exhibit antimicrobial function (Kim et al., 2007). The AMPs may exhibit antimicrobial effects through several pathways, but the most frequent mechanism of action involves the electrostatic interactions between positively charged AMPs and negative bacterial membranes (Zasloff, 2002). The quantitative mass spectroscopy of amnion/chorion extract has detected several ribonucleases [e.g., RNaseT2, RNaseK6, RNase7, RNase5 (angiogenin), RNase H2 subunit C, RNase pancreatic] and hydrolase components with antibacterial potential that destroy the biofilms (Yadav et al., 2017). The lysozyme secreted by epithelial cells of AM is capable of hydrolyzing the peptidoglycan backbone of bacterial cells, damaging their cell membrane, followed by lysis of bacteria (Yadav et al., 2017).

In a study, the antimicrobial activity of cryo-preserved AM (containing viable cells) was compared to the air-dried or freeze-thawed devitalized AM (with non-viable cells) counterpart (Mao et al., 2017). The results showed that the antimicrobial property of AM is governed by soluble antimicrobial factors secreted from endogenous viable cells, whereas non-viable cells in dehydrated devitalized AM do not participate in the synthesis of antimicrobial proteins. Recently, Tehrani et al. (2017) have reported that the exposure of AM to IL-1β may improve the secretion of antimicrobial peptides, including elafin, cathelicidin LL-37, HBD-2, and HBD-3. In addition to protein-based antimicrobial compounds existing in the AM, lactoferrin and hyaluronic acid (HA) components may also participate in anti-inflammatory and antibacterial responses (Zare-Bidaki et al., 2017).

### 3.3 Immunomodularity

The clinical application of AM has shown negligible immune response without acute rejection (Niknejad et al., 2008). This finding may be attributed to low expression of human leucocyte antigens (HLA) class I and no expression of HLA class II by epithelial cells (Hori et al., 2006). Herein, no expression of immunogenic markers, including CD86, CD40, CD80, HLA-A, HLA-B, HLA-D, and HLA-DR, are noticed which determines low immunogenicity (Magatti et al., 2018). The presence of immune-regulatory factors, e.g., HLA-G, IL-10, TGF-β, HGF, prostaglandin E2 (PGE2), and indoleamine 2,3-dioxygenase (IDO) are also responsible for its immune privilege through the suppressive effect on the proliferation of T cells and the CD8^+^ activity (Parolini et al., 2008; Silini et al., 2017). The HLA-G is an immunosuppressive molecule that interacts with the ILT4 receptor on monocytes and dendritic cells or the ILT2 receptor on various immune cells, such as natural killer cells, B-cells, monocytes, and dendritic cells (Wassmer and Berishvili, 2020). The interaction of HLA-G with different types of immune cells can in turn inhibit their proliferation, and immunoglobulin secretion, or attenuate their innate cytotoxicity (Kapasi et al., 2000; Selmani et al., 2008).

The cells existent in AM are highly capable of blocking immune responses by influencing immune cells’ function and secretion (Wassmer and Berishvili, 2020). For example, the conditioned medium extracted from AM has shown apoptotic effects on *in vitro* cultured neutrophil cells, whereas the direct *in vivo* administration of epithelial cells of AM exhibited a significant influence on decreasing the infiltration of neutrophil cells (Zhou et al., 2003; Tan et al., 2014). The transplantation of AM cells has shown an inhibiting effect on the infiltration of immune cells (e.g., T cells, monocytes, and macrophages) to the injured site, thereby reducing intense inflammatory responses (Liu et al., 2012). The immunosuppressive action of AM cells or conditioned media derived from AM is related to the bioactive modulators secreted from these cells and their ability to induce M2 macrophages over M1 macrophages (Magatti et al., 2017; Silini et al., 2017). The polarized forms of macrophages (i.e., M1 and M2) are known for their opposite actions in the tissue regeneration process, where the M1 macrophages boost the inflammatory phase of healing and M2 macrophages contribute to the regeneration process while showing some anti-inflammatory effects (Barboni et al., 2012). Magatti et al. have reported that conditioned media derived from *in vitro* culture of MSC cells of AM can impede the proliferation and differentiation of B lymphocytes through the action of prostanoids such as PGE2 (Magatti et al., 2020). The AM cells can also regulate the cytokine and chemokine secretion of immune dendritic cells to increase anti-inflammatory cytokines such as IL-10 or decrease the release of proinflammatory mediators, including IL-8, IL-12p70, TNF-α, and MIP-1α (Magatti et al., 2015). The immunosuppressive effect may also be linked to the expression of the Fas ligand by epithelial and mesenchymal cells (Chopra and Thomas, 2013).

### 3.4 Angiogenesis duality

Studies have demonstrated that AM is able to suppress neovascularization due to its anti-angiogenesis property (Shao et al., 2004). This anti-angiogenesis can be explained by the physical barrier effect that inhibits the permeation of pro-angiogenesis factors (Kobayashi et al., 2002). It can also happen as a result of ECM proteins, including collagen α2 (IV), laminin-1, laminin-5, fibronectin, collagen type VII, integrin 4, and integrin 6, which are known for their inhibition effect on corneal neovascularization (Fukuda et al., 1999). Moreover, the amniotic cells can secrete cytokines with an anti-angiogenesis effect, such as endostatin and thrombospondin-1 (Hossain et al., 2019; Bakhshandeh et al., 2021; de la Torre et al., 2021). Thrombospondin-1 is a potential anti-angiogenic peptide produced by mesenchymal cells (Ju et al., 2022), whereas endostatin is an anti-angiogenesis factor that prevents the growth of endothelial cells (Ghazani et al., 2022). Furthermore, some proteins, including collagen XVIII, IL-10, IL-1Ra, TIMP-1, TIMP-2, TIMP-3, and TIMP-4 exhibit anti-angiogenic activity (Hao et al., 2000). On the other hand, epithelial cells may play a role in mediating anti-angiogenic characteristics through the production of IL-1Ra, TIMP-3, and TIMP-4 (Parolini et al., 2009). For instance, the pigment epithelium-derived factor (PEDF) expressed by AM has been shown to act as a potent chemical inhibitor of angiogenesis (Shao et al., 2004).

The pro-angiogenic activity of AM has also been reported in the literature (Niknejad et al., 2013; Niknejad and Yazdanpanah, 2014; Yazdanpanah et al., 2015; Abbasi-Kangevari et al., 2019). Niknejad et al. (2013) have shown that the angiogenesis effect of AM is a side-dependent phenomenon. They have demonstrated that the vessel formation in a dorsal skinfold chamber rat model is enhanced when the AM is positioned epithelial side, whereas the vascularization is suppressed when the material is positioned stromal side. The pro-angiogenic effect of AM may originate from PDGF, VEGF, and angiogenin secreted by mesenchymal cells (Farhadihosseinabadi et al., 2018). On the other hand, fibronectin is able to promote angiogenesis by activating the ERK signaling pathway via interacting with PDGF, EGF, and bFGF (Hu et al., 2023). Tsai et al. (2007) have concluded that the upregulating the expression of integrin, platelet-endothelial cell adhesion molecule-1 (PECAM-1), and VE-cadherin adhesion molecules in the cultured endothelial cells make them a potential candidate as vascular grafts.

### 3.5 Anti-fibrosis

Fibrosis is a well-recognized hypertrophic pathological characteristic observed during the wound healing process that occurs due to excess secretion of fibroblast cells activated by TGF-β1 (Garrido et al., 2018). TGF-β1 stimulates fibrogenesis by encouraging the synthesis of ECM and the deposition of collagen by resident cells (Gonçalves et al., 2014). The anti-fibrotic characteristic of AM is attributed to its inhibition effect on the expression of TGF-β1 receptors in fibroblast cells (Arki et al., 2023). For example, the AM patch has been used in the treatment of liver fibrosis for reducing collagen deposition by down-regulating the pro-fibrotic factors, such as TGF-β1 and IL-1 (Sant’Anna et al., 2016). Studies also have shown that AM can downregulate the expression of apelin ligands in cirrhosis liver (Garrido et al., 2018). The paracrine signaling activated by the release of soluble factors from resident cells of AM tissue is effective in reducing the activity of pro-fibrotic and pro-inflammatory factors, such as TGF-β, IL-6, TNF-α, and PDGF accompanied by increasing the expression of anti-inflammatory cytokines, including IL-10 (Sant’Anna et al., 2016; Silini et al., 2015; Manuelpillai et al., 2010). A study by Mao et al. (2019) showed that condition media prepared from viable lyophilized AM (VLAM) tissue provides antifibrotic properties due to the presence of anti-fibrotic cytokines, such as HGF and IL-1β. The VLAM was also effective in reducing the expression of collagen I and α-SMA as pro-fibrotic factors.

The epithelial cells of AM can further participate in alleviating abnormal fibril arrangement by secreting anti-fibrotic factors, such as bone morphogenetic protein-7 (BMP-7), PGE2, and IL-10 (Andrewartha and Yeoh, 2019). Similarly, mesenchymal cells exhibit an anti-fibrotic effect by blocking the Wnt/β-catenin signaling via secretion of IGFBP-3, Dickkopf-1 (DKK-1), and Dickkopf-3 (DKK-3) (Liu et al., 2022). In addition, the conditioned medium released from mesenchymal cells of AM presents an antifibrotic effect by decreasing the levels of α-SMA and collagen I as well as increasing the MMP-2, MMP-9, and MMP-13 levels, which help degrade excess ECM (Fu et al., 2018).

### 3.6 Tissue regeneration and re-epithelialization

The basement membrane of AM is an appropriate matrix for encouraging the migration, adhesion, and differentiation of epithelial cells, which ultimately accelerates the re-epithelization of the wound site (Nazanin et al., 2022). The expression of various growth factors, such as TGF-α, EGF, KGF, bFGF, HGF, and GF receptors, including KGF-R and HGF-R, play major roles in wound healing and tissue regeneration (Koizumi et al., 2000). Various types of collagenous and non-collagenous proteins also significantly contribute to tissue repair by supporting cell activities (Murri et al., 2018). Altogether, these mentioned biochemical cues accelerate tissue regeneration by encouraging cell proliferation and angiogenesis (Chen et al., 2021).

## 4 Commercial products

Due to the outstanding therapeutic function of AM for treating wounds and regeneration of tissues, there are several international companies producing health products based on AM. The immune privilege of AM-derived stem cells, including epithelial and mesenchymal cells, through a low expression of class I antigens (HLA-A, B, C) and no expression of class II antigens (HLA-DR) have made it possible to commercialize AM-related products without decellularization (Srinivasan et al., 2020). Therefore, AM-based products are mostly dehydrated or cryo-preserved forms of amnion and seldom are decellularized (e.g., Biovance). To further enhance the mechanical stability and ease of handling, some of the AM-related products are composed of both layers of amnion and chorion together, including AmniEffect, EpiFix, AmniFix, AmniBurn, and AmnioExcel Plus. The AM-based commercial products and their structural features along with specific medical applications are summarized in Table 1. As mentioned in Table 1, some of these products have obtained health approval from the Food and Drug Administration (FDA) and the American Association of Tissue Banks (AATB). The other portion of these products are under clinical investigation to get required health confirmations or even in the very early stages of development. Nevertheless, the approved AM products are considered aseptic healthcare products that do not show adverse effects on the healing process of patients. However, when using these products, it is of great importance to meticulously follow the usage protocol of the manufacturer and pay attention to the storage notes. Moreover, the sterility of the product should not be compromised. Furthermore, the AM product is supposed to be placed from the epithelial side on the wound bed to encourage angiogenesis (Munoz-Torres et al., 2023). Among all AM products, there is only one product in gel form (i.e., AmnioBarrier) which is under development to be tested for clinical trials and is not on the market yet. However, since the regulations for assessing AM tissue-derived products are standardized and well-established by regulatory organizations in healthcare, the production of processed tissue products, such as AM gel hopefully seems to be possible in the near future.

**TABLE 1:** AM-based commercial products.

Product	Company	Features	Safety approval/references	Therapy
Grafix	Advanced wound management Smith+Nephew Consolidated Inc.	Cryopreserved placental membranes stored at −75°C to −85°C	NICE	Wound covering of acute and chronic wounds in head-to-toe locations
Grafix PL membrane	Advanced wound management Smith+Nephew Consolidated Inc.	Lyopreserved placental membranes stored at room temperature (RT)	NICE	Skin and wound care, surgical applications across multiple different specialties
Plurivest and Dermavest	AediCell Inc.	Freeze-dried pulverized decellularized amnion/chorion/placental disk/umbilical cord tissues, pressed into a sheet form, stored at RT	FDA (as a human tissue-based product under section 361 of the Public Health Service Act)	Deep and tunneled wounds, Partial and full-thickness wounds, Drainage wounds, Trauma Wounds (abrasions, lacerations, and skin tears), Second-degree burns, Diabetic ulcers, Pressure Ulcers, Venous Ulcers, Chronic vascular ulcers, Surgical (donor sites/graft post Mohs surgery, post laser surgery, podiatric)
AmnioBioGraft	Alamo Biologics Inc.	Single-layer patch-like tissue derived from amnion	AATB, U.S. FDA	Regenerative medicine, Wound management, Chronic and non-healing dermal wounds, Cutaneous wound care, Reconstructive medicine, Ocular, Injuries and reparative eye work, Burn Care
Allowrap DS	Allosource Inc.	Dual layer amnion with epithelial layer facing outwards on both sides of the graft, stored at RT	Samaniego (2011), Hinderland and Alan (2012)	Trauma, Orthopedics, Biological barrier after surgical repair
PalinGen Hdromembrane	Amnio Technology Inc.	Human amnion packaged in sterile saline	AATB, FDA	Full and partial-thickness, acute, and chronic wounds
PalinGen membrane	Amnio Technology Inc.	Air-dried human amnion	AATB, FDA	Full and partial-thickness, acute, and chronic wounds
PalinGen XPlus	Amnio Technology Inc.	Air-dried human amnion chemically crosslinked with glutaraldehyde	AATB, FDA	Wound covering
Clarix Flo	Amniox Medical Inc.	Injectable particulate human amnion and umbilical cord tissue	AATB, FDA	Regenerative injection therapy, Sports medicine
XWRAP	Applied Biologics Inc.	Processed amnion wrap, cover, or patch	—	Regenerative medicine
AmnioShield	Atec Spine Inc.	Dehydrated dual-sided amnion/chorion membranes	U.S. FDA	Chronic and scarred wounds, Wound barrier
AmnioBarrier	Biohealing Inc.	The gel form of the amnion, stored at RT	Under development to be tested in clinical trials	Preventative measures for the development of unwanted adhesions after cesarean section deliveries and small pelvic surgery, Gynecology
AmnioDerm	Biohealing Inc.	Lyophilized biological patch from human amnion	FDA (Lipový et al., 2021; Schmiedova et al., 2021)	Chronic and acute injuries, burns, venous leg ulcers, arterial skin ulcers, pressure ulcers, neuropathic skin ulcers, lymphedema ulcers
AmnioDisc	Biohealing Inc.	Lyophilized amnion	Passed phase 3 clinical trials, ready to enter the market	Eye and ear wounds, corneal and ear erosions, neurotrophic ulcerations, acute chemical/thermal burns, non-healing epithelial defects, post-infectious keratitis, Bullous keratopathy, repair of tympanic, membrane perforations
AmnioDrop	Biohealing Inc.	Lyophilized particulate amnion intended for resuspension, stored at RT	Under development to be tested in clinical trials	Accelerated regeneration after eye surgery
AmnioEye	Biohealing Inc.	Lyophilized amnion, stored at RM or deep frozen in medium and stored at −40°C to −80°C	FDA	Keratitis of various origins, Corneal erosion, and ulcers, Bullous keratopathy, Mechanical and chemical injury to the eye, Lysis or perforation of the cornea, In the treatment of fornix adhesions
AM-Nx	Biohealing Inc.	Cryopreserved patch of amnion, deep frozen with medium, stored at −40°C to −80°C	FDA	Neurosurgery, Decompressive craniectomy, craniotomy
AmnioGraft	BioTissue Inc.	Cryo-preserved amnion with devitalized cells, stored at −80°C to 4°C	U.S. FDA	Pterygium, Mechanical dry eye, Corneal defects, High-risk trabeculectomies, Leaking glaucoma blebs, Chemical burns, Stevens-Johnson Syndrome, Strabismus removal of tumors
AmnioGuard	BioTissue Inc.	Ultra-thick cryo-preserved amnion with devitalized cells, stored at −80°C to 4 °C	U.S. FDA	Shunt tube exposure prevention, Scleral melt/ischemia, Fornix and socket reconstruction, Marginal entropion repair, Removal of tumors or lesions, Symblepharon, Descemetocele or perforation, Ocular dermal wounds, Limbal tumors surface reconstruction
Clarix 100	BioTissue Inc.	Thin cryo-preserved amnion with devitalized cells, stored at −80°C to 4°C	U.S. FDA	Minimally invasive Achilles, Midfoot/forefoot fractures, Tendon/nerve repair, Ganglion cyst excision, Bunionectomy, Cheilectomy, Surgical barrier
Clarix 1K	BioTissue Inc.	Cryo-preserved amnion with devitalized cells, stored at −80°C to 4°C	U.S. FDA	Complex bone and joint reconstruction, Soft tissue repair and reconstruction, Nerve repair and decompression, Joint arthroplasty and arthrodesis, Cartilage repair, Fractures and non-unions, Traumatic wounds and reconstruction, Surgical wound healing and dehiscence
Neox 100	BioTissue Inc.	Thin cryo-preserved amnion with devitalized cells, stored at −80°C to 4°C	U.S. FDA	Shallow wounds or large-wound areas, including Diabetic foot ulcers, Chronic wounds, Dehisced wounds, Granulating/epithelializing wounds, Hypertrophic scars/keloids, Non/minimally exudating wounds, Pressure ulcers, Venous ulcers, Burns
Neox 1K	BioTissue Inc.	Cryo-preserved ultra-thick amnion with devitalized cells, stored at −80°C to 4°C	U.S. FDA	A wide array of wounds including Diabetic foot ulcers, Chronic wounds, Venous leg ulcers, Arterial ulcers, Pressure ulcers, Wound dehiscence, and Burns
Prokera	BioTissue Inc.	Cryo-preserved amnion with devitalized cells, stored at −80°C to 4°C	U.S. FDA	Damaged ocular surfaces, inflamed or scarred stroma
Biovance	Celularity Inc.	Decellularized dehydrated human amnion sheet, stored at RT	AABB, U.S. FDA	Partial- and full-thickness, acute and chronic wounds (such as traumatic and complex wounds, burns, surgical and Mohs surgery sites; and diabetic, venous, arterial, pressure, and other ulcers), including wounds with exposed tendon, muscle, bone, or other vital structures
Biovance 3L Ocular	Celularity Inc.	Three-layered decellularized dehydrated human amnion sheet with stromal surface facing outwards on both sides, stored at RT	AABB, U.S. FDA	Corneal and conjunctival injuries or defects, Corneal epithelial defects, Pterygium repair, Fornix reconstruction
AmnioClip Plus (AC^+^)	Deutsche Gesellschaft fur Gewebetransplantation (DGFG) Inc.	Cryopreserved amnion clamped in the ring system, stored at −60°C	Kotomin et al. (2015), Hofmann et al. (2021), Approved by the Paul Ehrlich Institute (PEI) and German Medicinal Products Acts	Dry eye syndrome, Persistent epithelial defects including neurotrophic corneal ulcers (on host cornea/corneal transplants), Reconstructions of conjunctival injuries (e.g., burns or chemical burns, perforating trauma), Pterygium surgeries, Symptomatic bullous keratopathy, High-risk keratoplasty for limbal stem cell deficiency
ViaShield	Globus Medical Inc.	Dual-layer human amnion patch (chorion-free)	Cunningham et al. (2019)	Wound barrier
AmnioExcel	Integra Life Sciences Inc.	Dehydrated human amnion tissue, stored at RT	AATB, U.S. FDA (Barr, 2014; Lintzeris et al., 2015; Abdo, 2016; Rosenblum, 2016; Snyder et al., 2016; Boyar and Galiczewski, 2018; Thompson et al., 2019; Tsai et al., 2021; Doucette et al., 2022)	Closing chronic wounds, Wound covering, Diabetic foot ulcers
AmnioExcel Plus	Integra Life Sciences Inc.	Three-layer dehydrated human amnion-chorion-amnion layers	AATB, U.S. FDA, (Bonvallet et al., 2022)	Wounds
AmnioMatrix	Integra Life Sciences Inc.	Cryopreserved suspension derived from amnion and components of amniotic fluid	U.S. FDA	Advanced wound care especially tunneling or deep wounds, repair, reconstruction, and replacement of tissue to aid in the closing of chronic wounds
BioDFence G3	Integra Life Sciences Inc.	Dehydrated three-layer amnion-chorion-amnion, stored at RT	U.S. FDA	Surgical reconstructions, wound management, Tissue barrier in soft tissue repair
BioDOptics	Integra Life Sciences Inc.	Dehydrated human amnion	AATB, U.S. FDA	Covering ocular surfaces
BioDRestore	Integra Life Sciences Inc.	Morselized, flowable amnion tissue processed with CryoPrime technique	AATB, U.S. FDA	Wound care
Ambio5	IOP Ophthalmics	Multi-layer dehydrated amnion (thickness ˃ 100 μm)	Grewal and Mahmoud (2016), Naxer et al. (2018)	Surgical regeneration of ocular surface including Fornix reconstruction, Symblepharon, Vast pterygium excision
AmbioDisk	IOP Ophthalmics	Dehydrated amnion	Choi and Jeon (2022)	Ocular surface diseases and disorders, Corneal erosions, Neurotrophic ulcerations, Acute chemical/thermal burns, Non-healing epithelial defects, Conditions associated with excessive dry eye, Post-infectious keratitis (herpetic, vernal, bacterial)
AmbioDry2	IOP Ophthalmics	Single-layer dehydrated amnion (thickness of 35 μm)	Chun et al. (2013)	Pterygium excision, Chemical and thermal burns, Corneal ulcers, Bullous keratopathy
Amniburn	MiMedx Inc.	Dehydrated human amnion/chorion membranes	AATB	Partial thickness and full-thickness acute and chronic wounds, Head/face, Hands, Genitals, Feet, Bone and tendon, Points of articulation
Amnieffect	MiMedx Inc.	Thick lyophilized human placenta-derived membrane comprised of amnion layer, intermediate layer, and chorion layer	AATB	Amputations, Complex incision management, Dehiscence repair, Tendon and ligament repair, Exposed bone or hardware, Flaps, Laminectomies, Minimally invasive surgeries, Hysterectomy, Endometriosis, Pilonidal cysts
Amnifix	MiMedx Inc.	Dehydrated human amnion/chorion membranes prepared in sheet, fenestrated, and wrap configurations	AATB	Surgical applications including debridement, surgical wounds, Dehiscence repair, Tendon and ligament repair, Myomectomies, Bunionectomies, Rotator cuff repair, Posterior lumbar interbody fusion, Total knee and shoulder arthroplasty, Limb salvage, Amputations, Pilonidal cysts, Port sites
Epieffect	MiMedx Inc.	Lyophilized human placenta-derived membrane comprised of an amnion layer, intermediate layer, and chorion layer	AATB	Acute and chronic wounds, Post debridement, Dehisced wounds, Diabetic foot ulcers, Venous leg ulcers, pressure ulcers, Mohs repair, Deep or tunneling wounds
EpiFix	MiMedx Inc.	Dehydrated human amnion/chorion membranes in both sheet and mesh fenestrated configurations, stored at RT	AATB, (Zelen et al., 2015; Zelen et al., 2016; Bianchi et al., 2018; Bianchi et al., 2019)	Acute and chronic wounds, Debridements, Dehisced wounds, Diabetic foot ulcers, Venous leg ulcers, pressure ulcers, Mohs repair
AmnioBand membrane	Mtf Biologics Inc.	Dehydrated human amnion and chorion membranes, stored at RT	U.S. FDA	Acute or chronic wound covering
AmnioBand viable membrane	Mtf Biologics Inc.	Cryopreserved viable human amnion	U.S. FDA	Protective covering for internal and external tissue defects including acute, chronic, and surgically created wounds
Affinity	Organogenesis Inc.	Fresh amnion containing living cells preserved using hypothermic technique, refrigerated storage at 1°C to 10°C	U.S. FDA, (McQuilling et al., 2017a; Serena et al., 2020)	Variety of soft tissue repair applications as a physical barrier to protect the site of repair, including Tendon repair, Cartilage and osteochondral defects, Acute and chronic wounds
NuCel	Organogenesis Inc.	Cryopreserved amniotic suspension consisting of ECM particles, amniotic fluid cells, various cytokines, and growth factors	U.S. FDA	Bone fusion, Tendon repair, Acute limb salvage, Acute wounds, and Burns
NuShield	Organogenesis Inc.	Dehydrated amnion-chorion membranes preserved with the LayerLoc method, stored at RT	U.S. FDA, (McQuilling et al., 2017b; McQuilling et al., 2019)	variety of soft tissue repair applications as a physical barrier to protect the site of repair, including Tendon repair, Spine adhesions, fibrosis, Acute and chronic wounds
AMIcare	Royan Stem Cell Technology Inc.	Dried amnion as wound dressing, stored at RT	Nouri et al. (2018), Nilforoushzadeh et al. (2019)	Chronic and acute wounds
AmnioDisc	SinaCell Inc.	Decellularized lyophilized human amnion, stored at RT	Iran FDA	Repair of eye epithelial defects, repair of conjunctival defects, repair of various injuries such as chemical or thermal burns, for eye surgeries such as glaucoma surgery, oculoplastic surgery, healing eye pain
AmnioSin	SinaCell Inc.	Decellularized human amnion, stored at −80°C	Iran FDA	Ocular surfaces, Chronic wounds, including diabetic foot ulcers and burns
CellAmnioSin	SinaCell Inc.	Sterilized fresh human amnion, stored at −80°C	Iran FDA	Chronic wounds, including diabetic foot ulcers and burns
OculoMatrix	Skybiologics Inc.	Thin amnion-only tissue preserved with HydraTek process (thickness of 45 μm), stored at ambient temperature (10°C to 30°C)	U.S. FDA	Ophthalmology
VisiDisc	Skybiologics Inc.	Thick amnion-chorion membranes preserved with HydraTek process (thickness of 200 μm), stored at ambient temperature (10°C to 30 °C)	U.S. FDA	Ophthalmology
WoundEx 45	Skybiologics Inc.	Dehydrated amnion (thickness of 45 μm)	U.S. FDA	Homologous use as a wound covering in the management of acute and chronic wounds.
WoundEx 200	Skybiologics Inc.	Dehydrated amnion-chorion membranes (thickness of 200 μm)	U.S. FDA	Homologous use as a wound covering in the management of acute and chronic wounds.
BioXclude	Snoasis Medical Inc.	Dehydrated human de-epithelialized amnion-chorion membranes	FDA	dental, endodontic, oral maxillofacial, and periodontal regenerative procedures as a barrier, conduit, connector, or cushion
SurGraft	Surgenex Inc.	Dehydrated amnion sheet, stored at ambient temperature	FDA	Chronic non-healing foot ulcers, including diabetic, pressure, and venous ulcers
		Five variations including (I) SurGraft: single layer amnion, (II) SurGraft XT: dual layer amnion, (III) SurGraft TL: triple layer amnion, (IV) SurGraft AC: dual layer amnion/chorion, (V) SurGraft ACA: triple layer amnion/chorion/amnion		
Sursight	Surgenex Inc.	Dehydrated single-layer amnion, stored at ambient temperature	FDA	Ocular repair and reconstruction procedures
AmnioELITE	Surgilogix Inc.	Human amnion-only tissue (chorion-free) and amniotic fluid components	U.S. FDA	Chronic wound repair
SXBarrier	Surgilogix Inc.	Human amnion in both dry (stored at ambient temperature) and wet (packaged in saline) forms	AATB, U.S. FDA	Open incisions or laparoscopy surgical system procedures, Wound covering
SXFluid	Surgilogix Inc.	Cryopreserved liquid format ground human amnion-only tissue (chorion-free) and amniotic fluid components, stored at −80°C ± 15°C	U.S. FDA	Tissue repair and wound healing
AlloGen	Vivex Biologics Inc.	Natural liquid matrix derived from amnion fluid	AATB, U.S. FDA	Cushion surface articulation within the joint capsule
Cygnus	Vivex Biologics Inc.	Five variations including (I) Solo: single layer amnion, (II) Dual: dual-layered amnion graft, (III) Matrix: a flexible amnion-intermediate-chorion layers, (IV) MAX: more thickness graft derived from the umbilical cord, (V) MAX XL: Fenestrated version of MAX	AATB, U.S. FDA	Soft tissue barrier and wound covering to repair underlying damaged tissue, such as acute and chronic wounds, including diabetic foot ulcers and venous ulcers, burn care, dermatology, and oral surgery
MiAmnion	Vivex Biologics Inc.	Three variations including (I) Single: only amnion layer, (II) Dual: double amnion layer, (III) Matrix: amnion and chorion layers	AATB, U.S. FDA	Soft tissue barrier and wound covering in numerous clinical applications, including spine and neurosurgery, foot and ankle, wound care, burn care, and dermatology
ViaGenex	Vivex Biologics Inc.	Three variations including (I) Matrix: amnion-intermediate-chorion layers, (II) MAX: Umbilical cord membrane, (III) Cryo MAX: Cryo-preserved umbilical cord membrane	AATB, U.S. FDA	Soft tissue barrier and wound covering in numerous clinical applications, including wound care, burn care, oral surgery, shoulder, nerves, knees, tendons, OB/GYN, and urology

## 5 Biomedical applications of AM-derived hydrogels

The therapeutic functionality has motivated researchers to process AM by various methods to prepare TE scaffolds (Fenelon et al., 2023). These methods involve using fresh AM sheets, dehydrated AM sheets, cryopreserved AM sheets, freeze-dried microporous scaffold, electrospun mat, 3D printed constructs, and pepsin-solubilized dAM-derived hydrogels (Figure 2B). The application of AM tissue or other types of AM-derived scaffolds has thoroughly been reviewed in recent studies (Dadkhah Tehrani et al., 2021). The main focus of the current work is exploring the development of AM-derived hydrogel and its widespread applications in TE and RM.

The preparation of hydrogels from the decellularized extracellular matrix (dECM) consists of 1) enzymatic digestion into monomeric protein components and 2) the temperature-induced gelation of pH-neutralized pre-gel solution (Saldin et al., 2017). In the first step, the cleavage of the terminal telopeptide region of collagen within dECM material by the pepsin enzyme enables digesting in dilute acidic solutions (Li et al., 2022). The removal of terminal peptides of collagen also attenuates the immunogenicity of the dECM-derived hydrogels (Lynn et al., 2004). In the second step, the reformation of intermolecular bonds within monomeric protein units forms a three-dimensional (3D) gel network (Johnson et al., 2011). The loss of water during incubation at physiologic temperature increases the entropy within the dECM pre-gel solution and subsequently forms aggregates of collagenous subunits in a so-called “self-assembly” process (Saldin et al., 2017).

**FIGURE 3:**
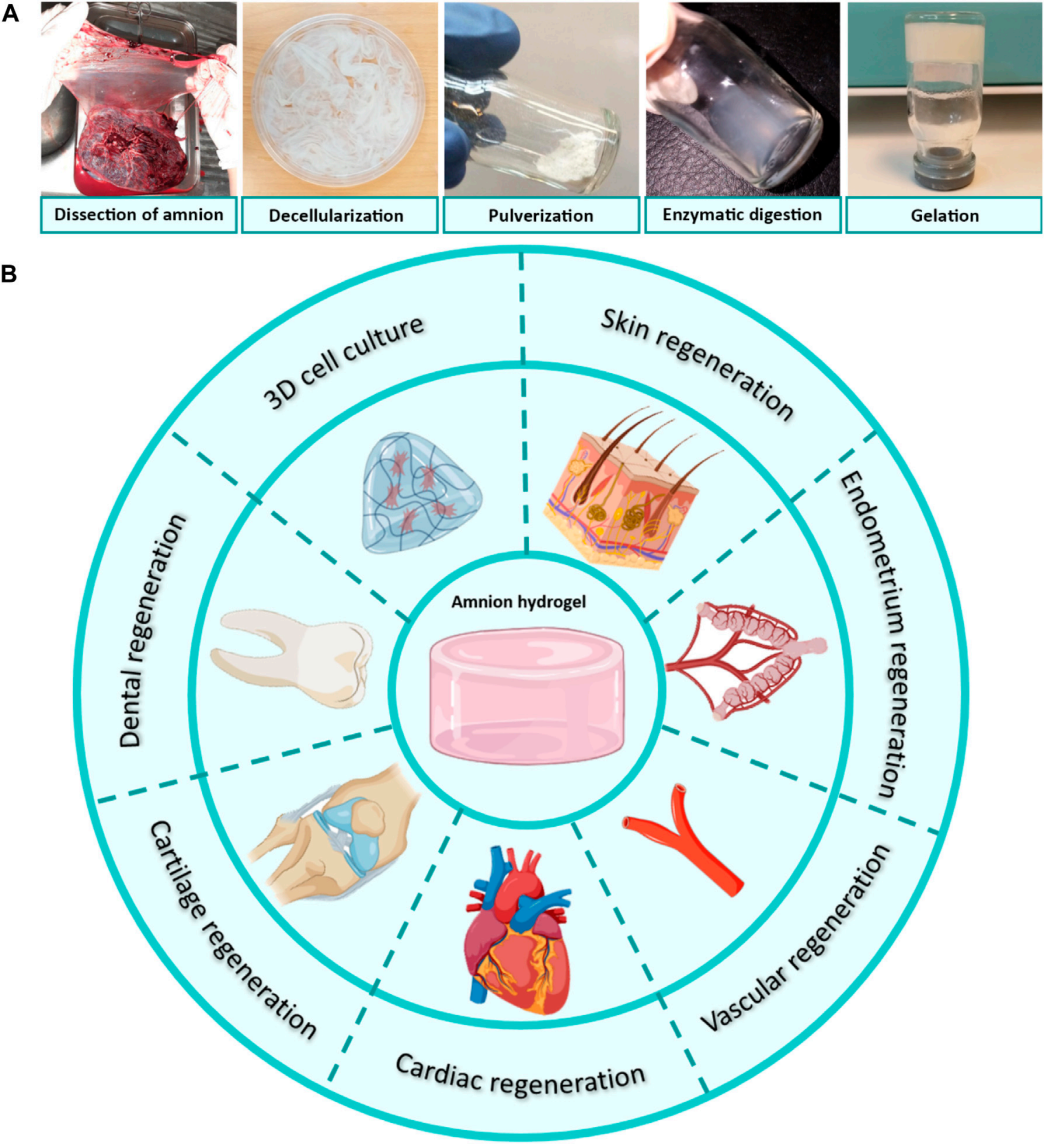
**(A)** Processing of dAM tissue into temperature-responsive hydrogels through dissection from placenta tissue, decellularization, lyophilization, pulverization, enzymatic digestion by pepsin in an acidic solution, and gel formation after incubation of pH-neutralized dAM solution at the physiologic temperature [Reprinted from Kafili et al. (2022), Copyright (2023), with permission from Elsevier]. **(B)** Tissue engineering applications of AM/dAM hydrogels. Panel 3B was partly generated using Servier Medical Art, provided by Servier, licensed under a Creative Commons Attribution 3.0 unported license.

The processing steps of AM tissue into a temperature-responsive injectable hydrogel include: 1) dissection of AM from underlying chorion tissue, 2) washing blood clots, 3) decellularization of AM tissue, 4) lyophilization and pulverization, 5) enzymatic digestion with pepsin, 6) incubation of pH-neutralized dAM solution at physiologic temperature (Kafili et al., 2022). Figure 3 shows these steps and the potential applications of the derived hydrogels. Processing AM into a hydrogel form has some advantages over the tissue form. The first benefit of AM hydrogel over AM tissue is related to the homogeneous distribution of biochemical cues in the hydrogel structure. Although the AM is considered a rich source of various proteins and bioactive growth factors, the distribution of these components varies in its different sublayers (Dadkhah Tehrani et al., 2021). This inhomogeneous distribution of the biomolecules may interfere with the aimed therapeutic characteristic (Jahanafrooz et al., 2023). For instance, the stromal side of AM exhibits better outcomes for wound healing applications as a result of EGF and TGF-β content (Gicquel et al., 2009), whilst the epithelial side shows promising results for ophthalmology surgeries due to its low content of TGF-β (Walkden, 2020). This medical choice is based on the fact that a high TGF-β content offers a stimulating effect on the over-deposition of collagen that may lead to hypertrophic scar formation and fibrosis, which in turn causes the loss of corneal transparency (Torricelli et al., 2016). Conversely, dAM hydrogels provide a more homogenous distribution of bioactive factors within 3D networks. On the other hand, despite the low immunogenicity of AM tissue, there is always a risk of inflammatory responses arising after transplantation (Gholipourmalekabadi et al., 2020). This issue can preferably be addressed by using decellularized AM tissue for the fabrication of injectable and temperature-sensitive hydrogels (Ryzhuk et al., 2018). Some of the other profits of dAM-derived hydrogel over AM or dAM tissues may include easier handling, better cell permeability, and control over tailoring mechanical properties by changing synthesis parameters, such as concentration, digestion time, etc. (Kafili et al., 2023a; Jahanafrooz et al., 2023).

The therapeutic properties of dAM hydrogels are multifaceted. So far, dAM hydrogels have been employed for the regeneration of the heart, skin, dental pulp, cartilage, vascular, fetal membrane, endometrium, and to a lesser extent for cell carrier vehicles (Figure 3B). It is noteworthy to mention that these studies are carried out as *in vitro* and *in vivo* setups. Considering the potential of AM tissue in wider biomedical applications, such as oral mucosa regeneration, bone repair, corneal regeneration, urinary bladder reconstruction, vaginoplasty, etc., its hydrogel has not yet been fully exploited. We believe that dAM hydrogels can be used in broader fields of medical therapies in the future, as it is reviewed in the following sections. A summary of recent advancements is shown in Table 2.

**TABLE 2:** Summary of application of AM/dAM hydrogels for various TE fields.

Tissue	Product	Experimental setting	Additive materials	Outcome	Ref.
Skin	Non-cellular hydrogel	*In vivo* (Immune compromised murine wound model)	Hyaluronic acid-based hydrogel (heprasil^a^, glycosil^b^, Gelin-S^c^, photoinitiator^d^, Extralink^e^)	• Acceleration of wound closure	Murphy et al. (2017)
		*In vitro* (HEKs and HDFs)		• Inhibition of wound contraction	
				• Promotion of neovascularization in wounds treated with HA-SAM hydrogel compared to HA hydrogel-treated or non-treated wounds	
Skin	Non-cellular gel	*In vivo* (Wistar rat skin with second-degree burn wounds)	Aloe vera (AV) extract, carboxymethyl cellulose sodium salt (CMC-Na) as a gelling agent, methylparaben as antibacterial ingredients, glycerin as a moisturizing agent, triethanolamine	• Less healing rate and angiogenesis	Rahman et al. (2019)
		*In vitro* (blood cells, HaCaT HFF1 cell lines		• More inflammatory response and scar formation in the AM/AV group compared to other treatment groups	
				• Enhanced re-epithelialization in the AM/AV group compared to other groups	
Skin	Non-cellular hydrogel	*In vivo* (Full-thickness Porcine skin)	Hyaluronic acid-based hydrogel (Heprasil, Gelin-S, Extralink)	• The complete healing, and normal pathological and histological results of wounds treated with HA-SAM hydrogel and AM powder	Murphy et al. (2020)
				• No intense immune rejection	
Skin	Non-cellular gel	*In vivo* (Wistar rat skin with second-degree burn wounds)	Rabbit’s skin collagen, CMC-Na as a gelling agent, methylparaben and propylparaben as antibacterial ingredients, glycerin as a moisturizing agent, triethanolamine	• Rapid wound healing; complete re-epithelialization	Rana et al. (2020)
		*In vitro* (blood cells)		• The higher wound contraction rate	
				• No histological observation	
Skin	Non-cellular scaffold	*In vivo* (Adult Wistar rats with full skin thickness ischemic excision with type 1 diabetes)	2-(N-morpholino) ethane sulfonic (MES), 1-ethyl-3-(3-dimethyl aminopropyl) carbo-diimide (EDC), N-hydroxy sulfosuccinimide (NHS) as crosslinking agents	• Higher thickness of newly formed epidermis and dermis	Nasiry et al. (2021)
				• More blood vessels	
				• Less inflammatory cells in dAM-derived scaffold-treated diabetic wounds compared to dAM membrane-treated or non-treated diabetic wounds	
Skin	Non-cellular hydrogel	*In vivo* (New Zealand rabbit with full-thickness epithelial tissue defect)	Methacrylated gelatin (GelMA), methacrylic anhydride (MA), and Acylphosphinate (AP) as the photoinitiator	• Supporting proliferation of fibroblast cells and expression of α-smooth muscle actin (α-SMA)	Zhang et al. (2021)
		*In vitro* (HFF)		• Acceleration of wound healing process	
				• Promotion of *in vivo* collagen deposition and angiogenesis	
Skin	Non-cellular hydrogel	*In vivo* (New Zealand rabbit with full-thickness skin defect model)	GelMA, MA, benzyl-2,4,6-trimethylbenzoylphosphinate (LAP) as photoinitiator	• Enhanced *in vitro* cell migration, angiogenic potential, and anti-inflammatory properties using AdECMMA-GelMA composite hydrogel	Chen et al. (2023)
		*In vitro* (RAW264.7 cells, HUVECs)		• Accelerated *in vivo* re-epithelialization	
Skin	Non-cellular injectable hydrogel	*In vitro* (L929 cell line)	Amine-terminated polyethylene glycol (AT-PEG), Laponite nanosilicates	• Improved distribution of ATPEG-modified nanosilicates in dAM hydrogel	Kafili et al. (2022)
				• Better cell adhesion to the hydrogel	
Skin	Non-cellular hydrogel bioink	*In vitro* (L929 cell line and HDFs)	Sodium alginate (Alg), Laponite nanosilicates	• Improved printability	Kafili et al. (2023b)
				• Enhanced cell proliferation	
				• Accelerated *in vitro* wound healing	
Skin	Non-cellular gel	*In vivo* (Wistar rat skin with second-degree burn wounds)	Titanium dioxide (TiO_2_) nanoparticles, carbopol 934 as a gelling agent, propylparaben, triethanolamine	• Antibacterial activity against *A.aureus*, *P.aeruginosa*, and *E.coli*	Islam et al. (2023)
				• Highest wound closure and faster re-epithelialization	
				• Lower inflammatory cell infiltration	
				• More vascular formation	
				• More collagen synthesis in wound area in the AM-TiO_2_ group compared to AM-only or TiO_2_ groups	
Skin	Non-cellular gel	*In vivo* (Wistar rats with second-degree burn wounds)	Silver nanoparticle, carbopol 934 gelling agent, acrylic acid, glycerine, triethanolamine	• Accelerated wound healing	Jhumi et al. (2023)
		*In vitro* cytotoxicity test (lethality test)		• Less inflammatory response	
				• Reduced epithelialization period in the AM-Silver group compared to AM-only or silver-only groups	
Cornea	Non-cellular in situ hydrogel/tablet/eye drop	In vivo (New Zealand rabbits with ocular acid burns)	Poloxamer 407 (P407), Polyvinyl alcohol (PVA)	• Less fibrosis and inflammatory responses in corneal burns treated with dAME containing P407 hydrogel	Luo et al. (2021)
		In vitro (CECs, CSCs, NIH3T3 fibroblasts)			
Cornea	Non-cellular hydrogel	In vivo (New Zealand rabbits with ocular alkali burn)	GelMA, LAP photoinitiator	• Prevention of symblepharon in the AME-GelMA eye pad treated ocular burns	Chen et al. (2020)
Heart	Non-cellular injectable hydrogel	*In vivo* (MI-induced Sprague-Dawley rats)	—	• Improved cardiac contractility	Henry et al. (2020)
		*In vitro* (BAECs)		• Decreased fibrosis	
				• Enhanced cardiac ejection fraction	
Vascular graft	Non-cellular hydrogel	*In vivo* (New Zealand white rabbits)	Alginate dialdehyde (ADA), EDC-NHS, Arg-Glu-Asp-Val (REDV) peptide, RGD peptide	• Supporting the attachment and proliferation of HUVECs	Peng et al. (2020)
		*In vitro* (HUVECs and HASMCs)		• Inhibiting the proliferation of HASMCs	
				• Inhibiting the aggregation and activation of platelets	
Vascular graft	Non-cellular hydrogel	*In vivo* (New Zealand rabbits with muscle incision)	Acrylamide, tetramethylethylenediamine (TEMED) as the catalyst, ammonium persulfate (APS) initiator, methylene bis acrylamide (MBAA) crosslinking agent, proanthocyanidin crosslinking agent, sodium alginate, SrCl_2_ crosslinking agent	• Anti-calcification ability	Lei et al. (2020)
				• High mechanical stability and elasticity	
		*In vitro* (HUVECs)		• Inhibiting the activation of platelets	
				• Enhancing the adhesion and proliferation of ECs	
Vascular	Cell-laden bioink	*In vitro* (NIH-3T3 and HUVECs)	Sodium alginate (Alg), CaCl_2_ crosslinking agent	• 3D bioprinting of large-scale pre-vascularized tissue with tubulogenesis	Heidari et al. (2023)
Vascularized tissue construct	Cell-laden bioink	*In vitro* (HSFs and HUVECs)	Methacrylic anhydride, Methacrylated hyaluronic acid (Hya-MA), LAP photoinitiator	• *In vitro* vasculogenesis in 3D bioprinted constructs	Comperat et al. (2023)
				• No significant difference between the biological capacity of methacrylated decellularized amnion (AdECMMA) or methacrylated decellularized chorion (CdECMMA) bioinks	
Cartilage	Cell-containing hydrogel	*In vitro* (bovine chondrocytes)	Fibrinogen, thrombin	• Secreting cartilage-specific ECM components, such as sGAG by chondrocytes encapsulated in AM-fibrin hydrogel	Hussin et al. (2011)
Cartilage	Cell-containing injectable hydrogel	*In vitro* (ADSCs and primary chondrocytes)	—	• The synergistic effects of dAM hydrogel and ADSCs in inhibiting catabolic response of IL-1β	Bhattacharjee et al. (2020)
				• Inhibiting Wnt/β-catenin signaling pathway	
Cartilage	Non-cellular hydrogel film	No *in vitro* or *in vivo* studies	Chitosan	• Enhancing the mechanical properties of chitosan by incorporating dAM hydrogel	Toniato et al. (2020)
Cartilage	Cell-containing injectable hydrogel	*In vivo* (Collagenase II-induced osteoarthritis (OA) rat model)	—	• The synergistic effect of ADSCs encapsulated in dAM hydrogel in mitigating the progression of OA by decreasing inflammation and activating regenerative pathways	Bhattacharjee et al. (2022)
Fetal membrane	Non-cellular 3D-printed medical device	*In vivo* (Pregnant swine model with damaged fetal membrane during fetoscopic surgery)	Polycaprolactone (PCL) framework	• Sealing fetal membrane defect	Lee et al. (2018)
		*In vitro* (NIH-3T3 cells and amnion MSCs)		• Preservation of amniotic fluid	
Uterine	Non-cellular injectable hydrogel	*In vivo* (Female Sprague Dawley Rat model of IUA)	—	• Fibrosis reduction of IUA	Li et al. (2022)
		*In vitro* (rEECs)		• Regeneration of endometrium	
				• Enhanced pregnancy rate	
Dental pulp	Both non-cellular and cell-loaded spongy scaffolds derived from dAM hydrogel	*In vivo* (Sprague-Dawley rats)	EDC-NHS crosslinking agents	• Mild to moderate inflammatory response after implantation	Bakhtiar et al. (2022)
		*In vitro* (hDPSCs)		• Revasculation of newly formed pulp tissue	
Dental pulp	Both non-cellular and cell-loaded hydrogel	*In vivo* (Sprague-Dawley rats)	Genipin crosslinking agent	• Filling the root canal using injectable dAM hydrogel	Bakhtiar et al. (2023)
		*In vitro* (hDPSCs)		• Low immunological responses	
				• Formation of pulp-like tissue with vascularization	
General tissue engineering/cell delivery	Cell-containing hydrogel	*In vivo* (Sprague-Dawley rats)	—	• Supporting cell viability for 3D cell culture	Ryzhuk et al. (2018)
		*In vitro* (PMSCs, BM-MSCs, C2C12, OECs)		• Less inflammation and immune response for dAM hydrogel compared to collagen hydrogel after 2 weeks of implantation	
3D cell culture	Both non-cellular and cell-loaded hydrogel	*In vitro* (hBM-MSCs)	Methacrylic anhydride (MA), Irgacure 2959 photoinitiator	• Versatile platform for 3D culture of cells	Deus et al. (2022)
				• Control over cell alignment by fabricating nano and micro topographical features on hydrogel surface	
Ovarian organs	Cell-containing hydrogel	*In vitro* (MEFs)	Alginate (Alg)	• 3D culture of oocytes	Haghshenas et al. (2022)
				• No significant difference in antral follicle formation between dAM-Alg hydrogel and Alg control group	

^a^ Heprasil is a thiolated hyaluronic acid with conjugated heparin groups.

^b^ Glycosil is a thiolated hyaluronic acid.

^c^ Gelin-S is a thiolated gelatin.

^d^ 2-hydroxy-4′-(2-hydroxyethoxy)-2-methylpropiophenone was used as the photoinitiator in Murphy et al. (2017) reference work.

^e^ Extralink is a polyethylene glycol diacrylate (PEGDA) crosslinker.

### 5.1 Tissue engineering and regeneration

The processing of AM/dAM into a hydrogel form has just become a focus of research in the last decade. However, even in such a short time, it has proven its therapeutic potential in the field of tissue engineering and regeneration of injured tissues for a broad range of tissue types. These biomedical applications of AM/dAM hydrogels based on target tissue are summarized in the following sections.

#### 5.1.1 Skin regeneration

Murphy et al. (2017) were among the pioneering research groups to develop amnion hydrogels by utilizing a photo-crosslinkable hyaluronic acid-based hydrogel containing solubilized amnion (HA-SAM). The HA-SAM liquid could be placed on the wound bed and crosslinked within several seconds of being exposed to UV light. An important feature was the utilization of native AM tissue instead of decellularized AM tissue. The solubilization process could kill the viable cells inside the AM tissue, inhibiting the immune rejection concerns. Further studies determined that the HA-SAM hydrogel provided accelerated wound closure, enhanced re-epithelialization, and higher density of small blood vessels compared to HA hydrogel-treated and non-treated wounds. The promoted neovascularization observed in wounds treated with HA-SAM hydrogel was most probably attributed to the presence of growth factors, such as fibroblast growth factor (FGF) family, epidermal growth factor receptor (EGF-R), and VEGF preserved within SAM (Cross and Claesson-Welsh, 2001). In another study, Murphy et al. (2020) showed that treating full-thickness wounds with HA-SAM hydrogel or AM powder resulted in faster wound closure rate, faster re-epithelialization, and minimum contraction compared to other treatment groups, including sterile bandage, HA hydrogel without SAM, and two commercial products of AmnioGraft (cryo-preserved amnion sheet) and Graftjacket (decellularized human dermis matrix). The better performance of AM-related products compared to other treatments could be attributed to the preservation of various biochemical cues (e.g., heparin sulfate, chondroitin sulfate, and other GAGs and proteoglycans) that regulate the regeneration of tissue (Murphy et al., 2020). The outcomes of skin regeneration by these AM products in the porcine model revealed the potential of these AM-derived products for translational medicine (Murphy et al., 2020). Zhang et al. (2021) applied a methacrylated gelatin (GelMA) layer over a methacrylated decellularized human AM (AdECMMA) to fabricate a photo-crosslinkable hydrogel for wound healing. This rationale design of the biomimetic bilayer was based on the mechanical support provided by the GelMA layer and the bioactivity provided by the AdECMMA component. Their results showed that the bilayer AdECMMA-GelMA skin substitute supported the transformation of fibroblasts into myofibroblasts and enhanced wound contraction during the wound-healing process. Furthermore, the bilayer scaffold exhibited promoting effects on collagen deposition and angiogenesis *in vivo*. These findings further elaborated the preference of the dAM-derived biomaterial compared to other prevalent biocompatible materials, such as GelMA, owing to its rich bioactive components (Zhang et al., 2021). Chen et al. (2023) have developed a composite hydrogel derived from a mixture of AdECMMA and GelMA for treating skin wounds. The wound healing process was improved accompanied by keratinization of the epidermal layer in the AdECMMA-GelMA group compared to the GelMA-only group. Interestingly, the infiltration of inflammatory cells in the tissue treated with composite hydrogel was significantly less than that in the wounds treated with GelMA-only hydrogel, which is likely due to intrinsic anti-inflammatory characteristic of dAM matrix (Chen et al., 2023). Nasiry et al. (2021) prepared three-dimensional (3D) microporous scaffolds by lyophilization of dAM hydrogels for the treatment of diabetic wounds. The engraftment of dAM-derived scaffolds into diabetic wounds caused upregulation of regeneration markers, including TGF-β, bFGF, and VEGF, and downregulation of pro-inflammatory cytokines, including TNF-α and IL-1β. The collagen deposition was also increased in diabetic wounds, which could be due to the presence of collagen fibers or stimulation of collagen synthesis by fibroblasts as a result of bFGF and TGF-β cytokines preserved in the dAM scaffold (Nasiry et al., 2021).

The insufficient printability of dAM-derived hydrogels is similar to other dECM-derived hydrogels due to their low viscosity and time-limiting temperature-sensitive gelation process (Kim et al., 2020). We have recently designed and developed a printable nanoengineered bioink based on dAM hydrogels for wound healing (Kafili et al., 2023b). We have shown that the employment of sodium alginate (Alg), as a structurally supportive component, in combination with dAM hydrogels provides enough stability for 3D printing of self-standing tubular constructs. We have also investigated the effect of Laponite nanosilicate as a physical crosslinker and rheology-modifying agent on the improvement of printing quality. Laponite is a disc-shaped two-dimensional nanoclay with a thickness of ∼1 nm and a diameter of about 25–30 nm (Kafili et al., 2023c). The anisotropic distribution of electric charges on the surface/edge of Laponite nanosilicates enables them to interact with various biopolymers non-covalently and strengthen the polymeric networks via the formation of physical crosslinks (Lokhande et al., 2018). Our results have affirmed that non-covalent electrostatic interactions between the positively charged edges of Laponite with anionic Alg or negatively charged surfaces of Laponite and amine groups of the dAM matrix improve the mechanical stability and rheological characteristics required for better printability (Kafili et al., 2023b). Nevertheless, nozzle clogging due to the formation of nanosilicate aggregates in ion-containing dAM solution was likely to occur when the Laponite concentration exceeded 2 %w/v. On the other hand, the addition of nanosilicates cooperates in the acceleration of cell proliferation and migration as a result of the bioactivity of ions degraded from the nanosilicates (Kafili et al., 2023b). In an attempt to improve the dispersibility of Laponite in dAM hydrogels, we have examined the effect of stabilization of nanosilicates by amine-terminated polyethylene glycol (AT-PEG) (Kafili et al., 2022). We have demonstrated that layer-by-layer self-assembly of collagen fibers and Laponite clusters occurs in Laponite-containing dAM hydrogels due to the disparate hydrophilic nature of nanosilicates and the dAM matrix. The coating of Laponite nanosilicates with the hydrophilic AT-PEG agent improves their distribution in the hydrophilic dAM matrix (Kafili et al., 2022). Therefore, dAM hydrogels containing PEG-modified nanosilicates are a potential candidate for TE applications, particularly for skin regeneration.

Rahman et al. (2019) have shown the advantageous effect of AM/Aloe vera (AV)/carboxymethyl cellulose sodium (CMC-Na) hydrogels for the medication of second-degree burns. Their results have determined that the healing rate of burn wounds treated with AV hydrogel is higher than that of AM-treated and AM/AV-treated wounds. However, more scar formation is noticeable after 4 weeks. Herein, the scar formation in the AM group is less than in the AV and AM/AV groups. The AM/AV group also exhibits a higher inflammatory score after 18 days of treatment. Therefore, it can be deduced that the synergistic effect of AM/AV facilitates the re-epithelialization process without affecting wound healing in terms of the inflammatory response, healing rate, remaining scar mark, and angiogenesis. Rana et al. (2020) have developed a hydrogel containing AM, collagen, and CMC-Na gelling agent for treating second-degree burn wounds. Their results indicate that the AM-containing hydrogel facilitates the healing process without a sign of scar formation and promotes re-epithelialization in a quicker time, as compared to the control groups (i.e., 1% silver sulfadiazine and the non-treated group as positive and negative control, respectively). Islam et al. (2023) have shown the positive effect of titanium dioxide (TiO_2_) nanoparticles (NPs) in AM gels containing Carbopol 934 (as a gelling agent) for the treatment of second-degree burn wounds. Their study reveals that treating burn wounds with AM-TiO_2_ gels enhances wound closure and re-epithelialization while encouraging vascular formation. Their results also exhibit less scar formation after the treatment of burn wounds, which is an important feature from the aesthetic point of care. Similarly, the antibacterial, anti-inflammatory, and anti-angiogenic properties of silver NPs for the treatment of burn wounds have been shown in several studies (Pourali and Yahyaei, 2016; Huang et al., 2022; Ajaykumar et al., 2023). For instance, Jhumi et al. (2023) have shown that the incorporation of Ag NPs into AM gels accelerates the healing of second-degree burn wounds in terms of expedited re-epithelialization and elevated contraction. It is noteworthy that in this study and many others (Rahman et al., 2019; Rana et al., 2020; Islam et al., 2023; Jhumi et al., 2023), AM tissue has been utilized without decellularization.

#### 5.1.2 Heart regeneration and treatment

Myocardial infarction (MI) is a heart failure-associated issue caused by blockage of blood flow to the heart followed by ischemia and tissue death (Kim et al., 2018). The disability of the myocardial tissue to regenerate itself after MI leads to scar formation during left ventricular (LV) regeneration and eventually heart failure (Tallquist and Molkentin, 2017). So far, various tissue engineering (TE)-based treatments, including stem cell therapies, cardiac patches, and injectable hydrogels, have been employed to improve cardiac regeneration after MI (Hashimoto et al., 2018). However, studies on the application of dAM-derived hydrogels for heart treatment are rare. The angiogenesis, antifibrotic, and anti-inflammatory properties of the dAM-derived matrix offer an exquisite alternative for the treatment of heart post-MI (Blume et al., 2021). In a recent study by Henry et al. (2020), the treatment of MI-induced myocardium using injectable dAM hydrogels has been demonstrated. They have shown that dAM-treated myocardium with acute infarction exhibits higher left ventricular ejection fraction (LVEF), enhanced fractional shortening, and reduced infarct size compared with the PBS-treated control group.

#### 5.1.3 Vascular tissue engineering

**FIGURE 4:**
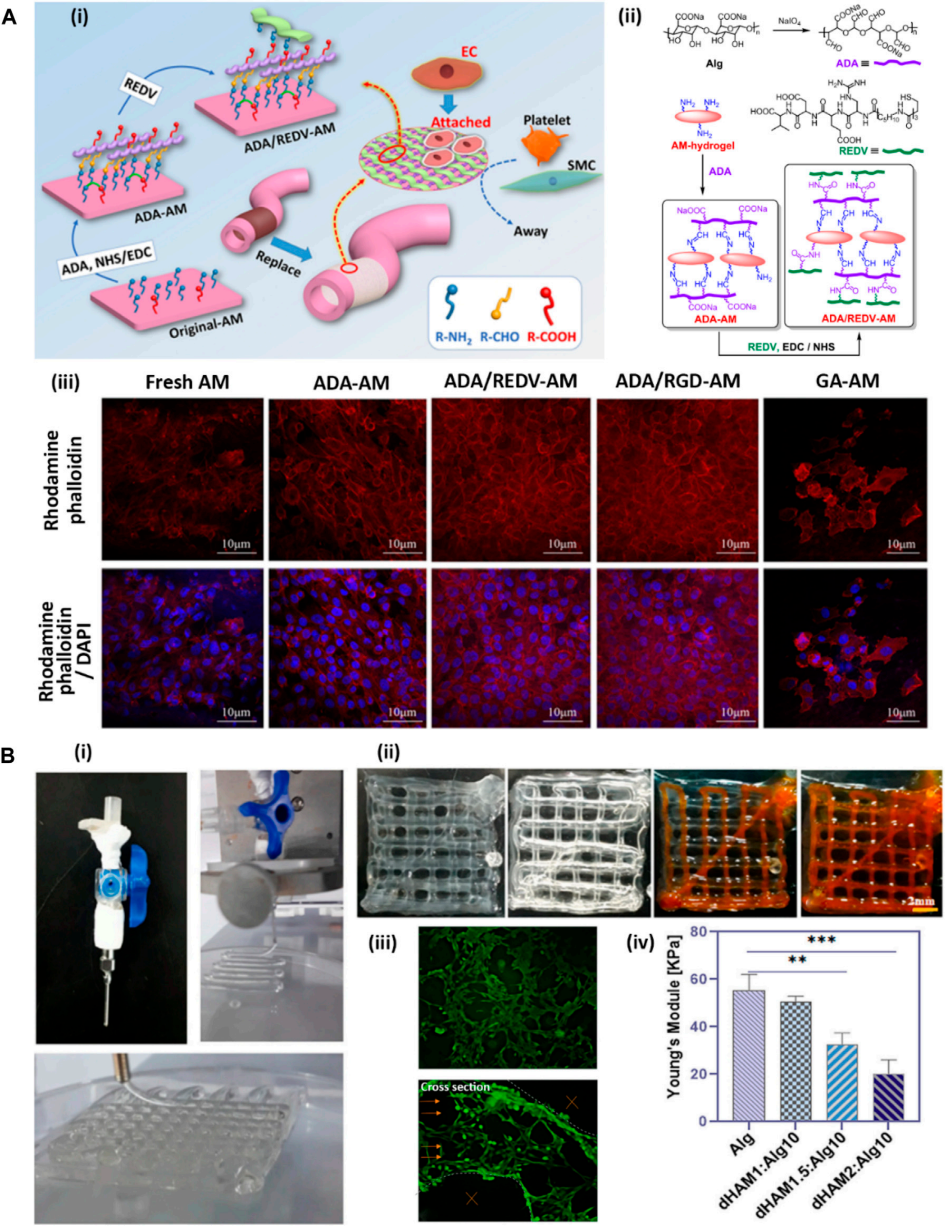
Development of AM-based hydrogels for vascular tissue engineering [adapted with permission from Peng et al. (2020), Copyright (2020) American Chemical Society]. **(A)** ADA-dAM hybrid hydrogel modified with REDV peptide as a vascular graft. **(A-i)** Representative image of modification steps of dAM hydrogel as an artificial vascular graft. **(A-ii)** Chemical bondings between dAM, ADA, and REDV peptide. **(A-iii)** CLSM images of HUVECs loaded in various dAM-based hydrogels, including fresh dAM, ADA-dAM, ADA/REDV-dAM, ADA/RGD-dAM, and GA-crosslinked dAM hydrogels stained with rhodamine-phalloidin for cytoskeleton organization and DAPI for cell nuclei. **(B)** Coaxial bioprinting of vascularized constructs [Reprinted from Heidari et al. (2023), Copyright (2023), with permission from Elsevier]. **(B-i)** Bioprinting of lattice structure using coaxial nozzle containing cell-loaded Alg-dAM particulate bioink as sheath and CaCl_2_ crosslinker solution as core material. **(B-ii)** Perfussability of the microchannels in 4-layer bioprinted construct exhibited through injection of orange dye. **(B-iii)** Live-dead staining of HUVECs in bioprinted microchannels and their cross section indicating tubulogenesis. **(B-iv)** Mechanical properties of lattice constructs printed using bioinks containing various amounts of dAM powder.

Cardiovascular diseases are among the common complications faced by modern societies that may lead to death in some cases (Newman et al., 2017). One of the main medical procedures for treating cardiovascular diseases is the replacement of the vasculature with artificial grafts (Adipurnama et al., 2017). Synthetic vascular grafts suffer from the occurrence of stenosis or thrombus after transplantation due to their intrinsic procoagulant property and low cell adhesion rate (Radke et al., 2018). Among various dECM-derived materials, dAM has attracted abundant attention for vascular replacement due to its biological properties, including cytocompatibility, anti-inflammation, anti-fibrosis, and low immunogenicity (Swim et al., 2019; Cheng et al., 2021; Wang et al., 2023). Nevertheless, the dAM sheet lacks the appropriate processability required for vascular reconstruction while its rapid biodegradability limits *in vivo* applications (Wehmeyer et al., 2015). Peng et al. (2020) have developed a dAM hydrogel-based biomaterial as an artificial vascular intima that mimics the structural and functional features of natural blood vessels (Figure 4Ai). The designed vascular graft is composed of a thermosensitive dAM hydrogel crosslinked with alginate dialdehyde (ADA) via chief-base reaction. The crosslinking is preceded by imine linkage between aldehyde groups of ADA and amine groups of dAM. The hydrogel is further grafted with Arg-Glu-Asp-Val polypeptide (REDV) via 1-ethyl-3-(3-dimethyl aminopropyl) carbo-diimide (EDC)/N-hydroxy sulfosuccinimide (NHS) catalysis to mimic the anticoagulant characteristic of natural blood vessels (Figure 4Aii). The REDV peptide is an endothelial cell (EC)-specific ligand that is commonly utilized for the modification of vascular substitutes because of its potential to stimulate rapid endothelialization while inhibiting platelet adhesion (Yang et al., 2015). As shown in Figure 4Aiii, this vascular graft shows selectivity for supporting the adhesion and proliferation of ECs while impeding the attachment and proliferation of smooth muscle cells (SMCs) (Peng et al., 2020). Implantation of the ADA/REDV-dAM vascular graft in rabbit models provides a template for EC growth along with hindering thrombosis occurrence. Lei et al. (2020) have developed a vascular graft based on dAM-polyacrylamide (PAM)-alginate (Alg) hydrogels to support the adhesion, proliferation, and migration of ECs while inhibiting the activation of platelets. This vascular graft enhances the secretion of nitrogen oxide (NO) and prostacyclin (PGI_2_) by HUVECs, which in turn play important roles in vascular remodeling. Besides, the graft exhibits an anti-calcification effect, which is presumably related to the presence of anti-inflammatory factors within the dAM matrix. These factors significantly downregulate the secretion of calcification-related proteins by inflammatory mediators, such as TNF-α and IL-6 (Pober and Sessa, 2015; Lei et al., 2020).

In a recent study, a cell-laden construct with vessel-like microchannels was 3D bioprinted using a coaxial nozzle with cell-encapsulated Alg bioink containing particulate dAM tissue as sheath and CaCl_2_ crosslinker solution as core material (Figure 4Bi) (Heidari et al., 2023). By changing the feed rate of core and sheath materials, perfusable microchannels with a thickness between 100 and 400 μm were successfully fabricated (Figure 4Bii). This study confirmed that HUVECs encapsulated in optimized bioink with 0.6 %w/v dAM particle content within the bioprinted construct were able to arrange themselves toward tube formation as a sign of the formation of blood vessels (Figure 4Biii). Therefore, these types of bioprinted constructs may have great potential for engineering pre-vascularized thick tissues. However, using the particulate dAM in the bioink formulation led to a declined elastic modulus of the bioprinted constructs as a result of weak chemical bonding between Alg and dAM particles (Figure 4Biv). Moreover, the high content of dAM particles in the bioink hydrogel caused reduced pore sizes by filling the pore areas within the bioprinted scaffold, which may harm nutrient transportation and cell activities, such as cell proliferation. Therefore, it seems that using the solubilized form of dAM as bioink can be considered a better alternative instead of the direct incorporation of dAM particles in the formulation to resolve the above-mentioned challenges (Lee et al., 2020). In another study done by Comperat et al. (2023), bioinks comprised of 0.3% methacrylated decellularized amnion (AdECMMA) or methacrylated decellularized chorion (CdECMMA) reinforced with 1.5% methacrylated hyaluronic acid (Hya-MA) was utilized for 3D bioprinting of vascularized explants. The matured tissue constructs bioprinted with HUVECs encapsulated in AdECMMA-HyaMA and CdECMMA-HyaMA bioinks demonstrated a better capillary-like structure organization and vasculogenic networks compared to HyaMA-only bioink as the control group. However, the incorporation of CdECMMA or AdECMMA in the bioink did not provide significantly different results for the *in vitro* maturation of ECs.

#### 5.1.4 Cartilage regeneration

Osteoarthritis (OA) is a disease condition of cartilage degeneration caused by inflammation induced by the expression of IL-1β and TNF-α pro-inflammatory cytokines (Heinegård and Saxne, 2011). Bhattacharjee et al. (2020) have employed a dAM hydrogel, as a platform for delivering adipose-derived stem cells (ADSCs), to decrease IL-1β induced inflammation in stimulated chondrocytes. ADSCs are well-known for their paracrine effects on inhibiting inflammation and down-trending cartilage degeneration through secreting anti-inflammatory factors (e.g., IL-10, IL-1RA, and TGF-β). On the other hand, the AM is capable of suppressing pro-inflammatory cytokines, including IL-1α and IL-1β, and decreasing the expression of matrix-degrading factors (MMPs) through the presence of natural MMP inhibitors within its matrix (Niknejad et al., 2008). Moreover, the elastase inhibitors, including elafin, SLPI, and β-defensin within AM, are responsible for their potential anti-inflammatory and antibacterial properties (King et al., 2007). Hence, dAM hydrogels for carrying ADSCs not only support the viability, proliferation, and stemness of stem cells but also provide a synergistic effect on reducing catabolic responses of inflamed chondrocytes (Bhattacharjee et al., 2020). In another study, Bhattacharjee et al. (2022) investigated the effect of intraarticular injecting of the dAM hydrogel with or without ADSCs into a collagenase-induced OA rat model. They have shown that both cell-free dAM hydrogels and ADSC suspensions provide comparable outcomes in decreasing inflammation-induced damage and promoting tissue regeneration in articular cartilage. Meanwhile, the encapsulation of ADSCs into dAM hydrogels synergetically enhances the therapeutic effect.

Chitosan, a linear polysaccharide derived from chitin, is well-recognized for its biocompatibility and antibacterial properties (Tohidi et al., 2022). The combination of chitosan with the dAM matrix containing various types of collagen (especially collagen type IV) offers an interesting choice of biomaterial for biomimicking the cartilage-related microenvironment (Toniato et al., 2020). Toniato et al. (2020) have developed a hybrid chitosan-dAM hydrogel for articular cartilage TE. Despite tailoring the mechanical properties by adjusting the concentration of dAM (2.5, 5, and 10%w/v) at constant chitosan concentration (2%w/v), the highest obtained elastic modulus (i.e., ∼80 kPa for 2% chitosan-5% dAM hydrogel) is still inferior to that of native articular cartilage [500–2000 kPa (Zhou et al., 2018)]. Therefore, chitosan-dAM hydrogels require further mechanical support to better mimic the cartilage tissue regarding mechanical properties. Hussin et al. (2011) have fabricated 3D scaffolds for cartilage TE by incorporating the HAM particulates (without decellularization) into fibrin hydrogels. The resemblance of the chondrocyte-embedded fibrin-HAM hydrogel to hyaline cartilage with the capability of secreting sulfated GAG has been demonstrated.

#### 5.1.5 Endometrium and fetal membrane regeneration

**FIGURE 5:**
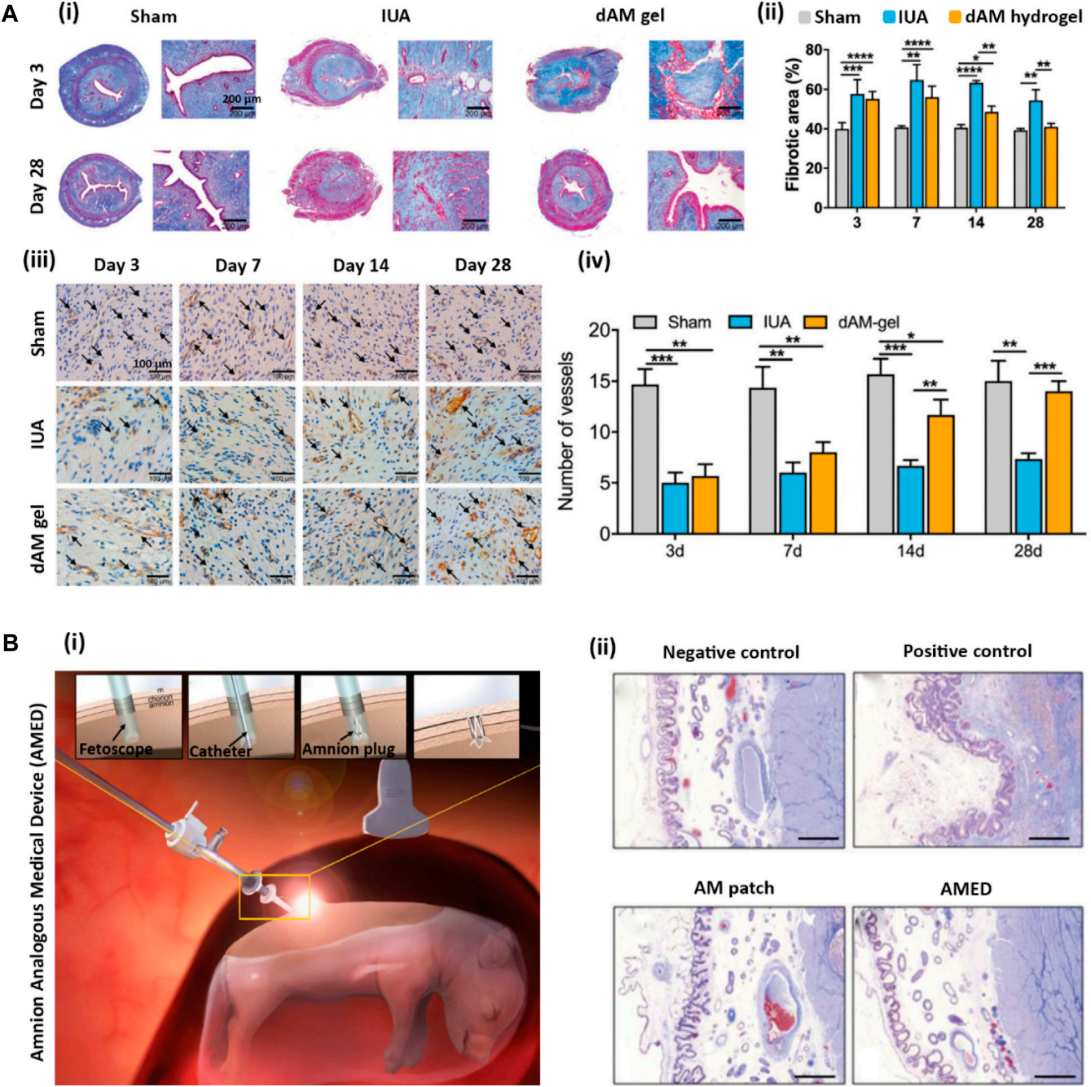
**(A)** Endometrium regeneration using dAM hydrogel [Reprinted from Li et al. (2022), Copyright (2022), with permission from Royal Society of Chemistry]. **(A-i)** Histological Masson’s trichrome staining of uterine after 3–28 days after operation in the sham operation group (Sham), endometrium damage group (IUA), and dAM hydrogel treated group (dAM gel); **(A-ii)** Quantification of fibrotic area in uterine treated from different groups; **(A-iii)** IHC staining of CD31 at 3, 7, 14, and 28 days post-operation (Black arrows show the capillaries); **(A-iv)** quantification of number of blood vessels. **(B)** Healing of fetal membrane using developed decellularized amnion analogous medical device (AMED) [Reprinted from Lee et al. (2018), Copyright (2018), with permission from Wiley]. **(B-i)** Fetoscopic surgery procedure using inserted AMED to fill the amniotic cavity; **(B-ii)** Histological Masson’s trichrome staining of *en bloc* sections of uterine in negative control (unmanipulated sac), positive control (unclosed entry site), and fetoscopic access site covered with commercial AmnioGraft patch and with AMED.

Intrauterine adhesion (IUA), also known as Asherman syndrome (AS), is a uterus disease situation related to the formation of fibrotic tissue in the uterine cavity as a result of damage to the endometrium (Hooker et al., 2014). Decellularized and lyophilized AM is one of the ECM-derived biomaterials that have been utilized clinically for treating intrauterine damage by suppressing TGF-β1 (which is responsible for developing IUA) (Chen et al., 2018). However, the fresh dAM membrane is very difficult to fix and suture into the uterus cavity. Hence, processing dAM into hydrogels provides the opportunity to fill irregularly shaped cavities. Islam et al. (2023) have recently employed a thermosensitive dAM hydrogel to prevent IUA in rat models. It has been shown that the injection of the dAM hydrogel into the uterus cavity can significantly decrease fibrosis formation through re-epithelialization of the damaged endometrium (Figures 5Ai, ii), while supporting angiogenesis within the regenerated endometrium (Figures 5Aiii, iv).

The rupture of fetal membranes during pregnancy is a serious issue that endangers the fetus and leads to premature delivery (Chmait et al., 2017). Lee et al. (2018) have designed a 3D-printed dAM-analogous medical device (AMED) comprising a polycaprolactone (PCL) framework filled with a dAM hydrogel for treating fetal membrane defects (Figure 5Bi). The fabricated AMED exhibits surgical handling privilege along with biocompatibility, non-immunogenicity, and proper healing of AM compared to other commercial equivalents (such as AmnioGraft patch, Bio-Tissue Inc.) to seal the fetal membrane and block the leakage of amniotic fluid (Figure 5Bii). As the developed AMED is easy to apply by a fetoscopic instrument, the device significantly decreases the surgical time in comparison with the transplantation of AmnioGraft.

#### 5.1.6 Dental pulp regeneration

An appropriate scaffold for endodontic regeneration requires biocompatibility to support the growth, proliferation, and differentiation of cells to the odontogenic lineage (Yuan et al., 2011). So far, various types of scaffolds using natural, synthetic, and composite biomaterials have been utilized for the restoration of endodontic functions (Moussa and Aparicio, 2019). Among the various types of scaffolds, injectable hydrogels are a preferable option to fill the canal roots with complex geometry (Chang et al., 2017). The dAM tissue as a biological scaffold is considered a promising candidate for dental pulp regeneration, mostly owing to its angiogenic and anti-inflammatory properties along with its accessibility (Honjo et al., 2015). Bakhtiar et al. (2022) have investigated the potential of spongy scaffolds derived from dAM hydrogels for regenerative endodontics. They have shown that the subcutaneous implantation of scaffolds derived from dAM hydrogels can cause mild to moderate inflammatory responses as evidenced by the migration of mononuclear inflammatory cells, such as lymphocytes and macrophages, and primary multinucleated giant cells to the implanted scaffolds. Interestingly, both the cell-free scaffolds and human dental pulp stem cells (hDPSCs)-loaded scaffolds have exhibited pulp-like tissue growth in the root canal area accompanied by angiogenesis after 7 weeks of implantation. The observed regeneration capacity is attributed to the presence of bioactive molecules, including bFGF and TGF-β, in the dAM matrix (Kim et al., 2012). However, a mild fibrosis formation has also been observed (Bakhtiar et al., 2022). In another study, pulp regeneration has been studied by injectable dAM hydrogels with or without hDPSCs (Bakhtiar et al., 2023). To reduce the biodegradability of the hydrogel, Genipin (up to 10 mM) was utilized to react with free amine residues of dECM (Nagaoka et al., 2014; Výborný et al., 2019). *In vivo* studies on the tissue regeneration in the root canal model implanted in rat calvaria have revealed that the hDPSC-seeded dAM hydrogels do not cause a significant difference in pulp-like tissue formation compared to cell-free counterparts. Therefore, the pulp-like tissue regeneration is likely attributed to the migration of housing calvarial progenitor cells instead of the odontogenic differentiation of hDPSC cells. No significant differences in infiltration of inflammatory cells, angiogenesis, and fibrosis have also been noticed. In summary, the dAM hydrogels seem to be useful for the formation of vascularized pulp-like tissue without sufficient effect on the formation of tubular dentin structure (Bakhtiar et al., 2023).

#### 5.1.7 Ocular regeneration

Both corneal tissue and AM share some mutual characteristics like thin thickness, being avascular, and proper light transmittance (Deihim et al., 2016). Therefore, AM has a long history of ocular transplantation for treating corneal injuries or diseases by promoting re-epithelialization, reducing scar formation and neovascularization, and controlling inflammatory responses (Pogozhykh et al., 2020). However, AM transplantation is prone to dissolving and rapid degradation that burdens multiple painful surgeries and might cause allergic reactions in some cases as an allograft (Shimazaki et al., 2004). Therefore, processing dAM into *in situ* forming hydrogels seems to be a practical solution to address these issues.

Yazdanpanah et al. have shown that thermoresponsive corneal dECM (dCO)-derived hydrogel possesses proper transparency as well as bioactivity to be used in ocular TE applications, owing to the presence of essential growth factors and proteins, such as collagen, lumican, keratocan, and laminin within corneal dECM (Yazdanpanah et al., 2021). They also compared the ECM composition of dCO with dAM and showed that these two tissue matrices share a great portion of similar proteins. Moreover, the dAM contained proteins like decorin and periostin, which are well-known for their therapeutic effects on the cornea (Yazdanpanah et al., 2021; R Mohan et al., 2011). These comparisons pave the way for the application of dAM hydrogel for ocular regeneration as well. We even predict that thermoresponsive dAM hydrogel can even provide better dynamic mechanical properties compared to dCO hydrogel at the same concentration by comparing available data in the literature [e.g., G′∼ 40 Pa for dCO hydrogel (Yazdanpanah et al., 2021) as compared to G′∼1.5 kPa for dAM hydrogel (Li et al., 2022) at the same concentration of 15 mg.mL^−1^]. The higher mechanical strength of dAM hydrogel compared to dCO hydrogel can enhance the structural stability of hydrogel upon application on ocular defects. However, to the best of our knowledge, there is no publication about the application of dAM hydrogels for ocular regeneration or TE, yet. Nevertheless, the incorporation of AM extract into various hydrogel formulations for treating ocular diseases has been exploited in several cases. For example, Luo et al. (2021) proposed the application of dAM extract (dAME) in different ways, such as eye drops, and the incorporation of dAME in temperature-sensitive P407 hydrogels or freeze-dried PVA tablets to treat corneas with acid burns. Treatment of severe corneal burns with these three alternatives was accompanied by the best results in terms of less stromal opacity and faster corneal regeneration for *in situ* forming dAME containing P407 hydrogel, followed by second best results for dAME containing PVA tablets, and moderate results for dAME liquid drop. The better therapeutic effect of dAME incorporated into a hydrogel can be due to sustained and long-term release of dAME compared to two other administration methods that improve its efficiency and reduce its loss during treatment. Moreover, the mRNA expression of TGF-β1 in corneal burns treated with hydrogel and tablet containing dAME was less than that in dAME liquid drop-treated and control groups, which impedes excessive stromal fibrosis. During the inflammatory phase of healing, the corneas treated with dAME incorporated hydrogel and tablet expressed higher LRIG1, which is a protein in corneal cells that prevents inflammatory responses and contributes to corneal transparency. In another study, eye pads were fabricated by loading AM extract (AME) in GelMA hydrogel and were used for treating ocular chemical injuries (Chen et al., 2020). The AME-GelMA eye pads showed similar re-epithelialization compared to AM transplantation, while the expression of TGF-β1 in AME-GelMA treated groups was lower than that in AM transplanted and control groups leading to the occurrence of less scar hyperplasia. In addition to these therapeutic effects, the AME-loaded GelMA eye pads have the advantage of processability and easier handling over AM tissue and can be fabricated in a dome shape to fit the conjunctival sac.

### 5.2 Cell therapy and cellular studies

In addition to the therapeutic effect of AM hydrogel for various TE and regenerative applications, it can further be utilized for other biological applications. For instance, AM hydrogel has shown excellent potential as a cell carrier for stem cell therapy (Ryzhuk et al., 2018). Moreover, it has the potential to be used as an *in vitro* platform to study cell behaviors, such as cell-matrix interactions, and the mechanistic effect of matrix on cell phenotypes, or be applied for disease modeling and drug screening purposes (Deus et al., 2022). Accordingly, these applications are discussed in the following sub-section.

#### 5.2.1 Cell carrier and cell culture platform

During the last decade, the delivery of stem cells to injured sites has gained great attention and has been applied in several pre-clinical and clinical experiments (Margiana et al., 2022). Stem cells are known to regulate the regeneration process of injured tissues by secreting various types of cytokines and growth factors (Murphy et al., 2013). However, the delivery of cells into the target tissue still suffers from insufficient cell survival and cell engraftment after transplantation. For instance, mesenchymal stem cells (MSCs) are required to adhere and spread in the desired environment to survive, thereby cell suspension delivery significantly degrades their integration into the target tissue (Phinney and Prockop, 2007). Systemic delivery of stem cells also faces serious issues related to their entrapment in the capillaries of untargeted tissues (Kraitchman et al., 2005). Therefore, the delivery of stem cells in a biocompatible carrier into the desired defect site can ensure their viability and survival after transplantation (Slaughter et al., 2009). Ryzhuk et al. (2018) have shown the potential of dAM hydrogels for the delivery of different cell sources, including placenta-derived mesenchymal stem cells (PMSCs), by supporting their viability and biological functionality. The implantation of dAM hydrogel in the back of rats exhibits less immune cell infiltration as compared to collagen hydrogel. The lower density of CCR7+ cells (macrophage marker) in dAM hydrogel compared to collagen hydrogel further affirms less immune reaction. Deus et al. (2022) have developed photo-crosslinkable methacrylated decellularized amnion (AdECMMA) hydrogels as a versatile platform for cell culture and TE. The results demonstrate that the degree of methacrylation has a significant effect on the morphology and viability of the encapsulated hBM-MSCs due to variations in the viscoelastic properties of hydrogels and their structural porosity. Micro- and nano-topographical grooves were made on the surface of the hydrogels and used as a guidance pattern for hBM-MSCs alignment. It has also been shown that both micro- and nano-features are very effective on cell spreading as compared to the hydrogel without topographical features. Haghshenas et al. (2022) have developed dAM-Alg hybrid hydrogels containing various amounts of dAM (15 mg/mL, 30 mg/mL, 45 mg/mL) for the 3D culture of oocytes. Although the developed hydrogels are efficient carriers for long-term culture of oocytes, no significant differences in antral formation rate, follicle diameter, and estradiol secretion are noticed as compared to the control group (Alg-only hydrogel). This inferior functionality is attributed to the loss of growth factors, such as bFGF and EGF, after decellularization of AM using sodium dodecyl sulfate (SDS) that are known to improve follicle growth (Price, 2016). Hence, the employment of other agents for the decellularization process of AM should be considered for future studies to better preserve biochemical cues within the dAM matrix for delivering oocytes.

## 6 Conclusion and future prospectives

The AM tissue has a long history in the medical field and has already been approved for clinical applications, such as treating burn wounds and corneal regeneration. There are also several commercial AM-related products for clinical use (Nejad et al., 2021). During the last decade, AM/dAM hydrogels have shown wonderful therapeutic outcomes for the regeneration of damaged tissues (Dadkhah Tehrani et al., 2021). Therefore, clinical applications of AM/dAM hydrogels are a rational and possible trend. Nevertheless, clinicians and researchers need to be more focused on examining the function of AM/dAM hydrogels on large animals and standardizing it for clinical applications to validate its safety and facilitate the approval processes.

Hydrogels derived from AM are considered a valuable platform for various medical therapies, owing to their reservoir of active biochemical cues and excellent bio-functionality (Deus et al., 2022). However, multiple processing steps, including decellularization and enzymatic digestion, can lead to the loss of some ECM components and alleviated mechanical stability (Sasikumar et al., 2019). It has been hypothesized that the utilization of micronized AM inside a hydrogel formulation can address this issue (Heidari et al., 2023). However, these materials may suffer from the inhomogeneous distribution of bioactive AM particles in the hydrogel matrix, interfering with expected therapeutic results. Processing AM/dAM into a hydrogel form or utilizing AM/dAM extracts can be considered practical alternatives to resolve these issues. Besides, the hydrogel derived from dAM privilege over AM hydrogel in the concept of thermo-sensitivity for minimally invasive therapies (Li et al., 2022).

Despite the promising medical outcomes of dAM hydrogels, their low mechanical properties and rapid degradability are some issues that need to be addressed. Several trending methods, such as crosslinking and modifying the hydrogel matrix with methacrylate groups for photo-crosslinking, may be helpful for the reinforcement of dAM hydrogel networks (Deus et al., 2022; Bakhtiar et al., 2023). Moreover, the combination of nanotechnology approaches with dAM hydrogel can be considered a beneficial tool in several biomedical directions. For instance, the incorporation of gold nanorods in dAM hydrogels can be considered for improving electrical conductivity and enhancing the functionality and maturation of cardiomyocyte cells (Zhang et al., 2019). The incorporation of TiO_2_ and silver NPs can offer antibacterial properties for treating infected or burn wounds as well (Islam et al., 2023; Jhumi et al., 2023). Other biocompatible nanomaterials, such as layered nanosilicates, are also usable for enhancing mechanical stability and printability (Kafili et al., 2023b). These kinds of examples seem to be endless and worth applying to provide superior therapeutic outcomes for AM/dAM hydrogels. Meanwhile, it is of great importance to precisely explore the effectiveness and biosafety of modified AM/dAM hydrogels before *in vivo* and pre-clinical experiments.

3D bioprinting is a powerful and reliable technique that facilitates controlling the internal structure and bulk dimensions of designed scaffolds (Abaci and Guvendiren, 2020). This fabrication method also provides control over the deposition of desired biomaterial or intended cell types on the exact pre-defined location, which expands the medical relevance of scaffolds by mimicking native biosystems (De Santis et al., 2021). It has been a while since dECM-derived bioinks have proven their priority for TE and RM, owing to their intrinsic biochemical cues, such as growth factors, cytokines, glycosaminoglycans, and regulatory proteins (Choudhury et al., 2018). Nevertheless, the application of dAM bioinks for 3D bioprinting has not been widely explored yet except few studies (Lee et al., 2018; Kafili et al., 2023b; Comperat et al., 2023). The preparation of bioinks based on dAM hydrogels is a huge breakthrough, which broadens its potential for regenerative therapy.

## Figures and Tables

**Table:** 

**AABB**	American Association Blood Bank
**AATB**	American Association of Tissue Banks
**ADA**	Alginate dialdehyde
**ADSCs**	adipose-derived stem cells
**Alg**	alginate
**α-SMA**	Alpha-smooth muscle actin
**AM**	Amnion
**AME**	amnion extract
**AMED**	amnion-analogous medical device
**AdECMMA**	methacrylated decellularized amnion
**AMP**	antimicrobial peptides
**AP**	Acylphosphinate
**APS**	ammonium persulfate
**AS**	Asherman syndrome
**AT-PEG**	amine-terminated polyethylene glycol
**AV**	Aloe vera
**BAECs**	bovine aortas endothelial cells
**bFGF**	basic fibroblast growth factor
**BM-MSCs**	bone marrow-derived mesenchymal stem cells
**BMP-7**	bone morphogenetic protein-7
**CECs**	Corneal epithelial cells
**CdECMMA**	methacrylated decellularized chorion
**CLSM**	Confocal laser scanning microscopy
**CMC-Na**	carboxymethyl cellulose sodium salt
**CSCs**	Corneal stromal cells)
**3D**	three-dimensional
**dAM**	decellularized amnion
**dAME**	decellularized amnion extract
**DAPI**	4′,6-diamidino-2-phenylindole
**dCO**	corneal decellularized extracellular matrix
**dECM**	decellularized extracellular matrix
**DKK-1**	Dickkopf-1
**ECM**	extracellular matrix
**ECs**	endothelial cells
**EDC**	1-ethyl-3-(3-dimethyl aminopropyl) carbo-diimide
**EECs**	endometrial epithelial cells
**EGF**	epidermal growth factor
**EGF-R**	epidermal growth factor receptor
**EG-VEGF**	endocrine gland-derived vascular endothelial growth factor
**FDA**	Food and Drug Administration
**FGF**	fibroblast growth factor
**GA**	glutaraldehyde
**GAG**	glycosaminoglycan
**GelMA**	methacrylated gelatin
**GF**	Growth factors
**HA**	Hyaluronic acid
**HAM**	human amnion
**HASMCs**	human aortic smooth muscle cells
**HBD**	human beta-defensin
**hBM-MSCs**	human bone marrow-derived mesenchymal stem cells
**HDF**	human dermal fibroblast
**hDPSCs**	human dental pulp stem cells
**H&E**	Hematoxylin and eosin
**HEKs**	human epidermal keratinocytes
**HFF**	human fetal fore-skin derived fibroblast
**HGF**	hepatocyte growth factor
**HGF-R**	hepatocyte growth factor receptor
**HLA**	human leucocyte antigens
**HSF**	human skin fibroblasts
**Hya-MA**	methacrylated hyaluronic acid
**HUVECs**	human umbilical vein endothelial cells
**IDO**	indoleamine 2,3-dioxygenase
**IFN-γ**	interferon-γ
**IGF**	insulin-like growth factors
**IGFBP-1**	insulin-like growth factor-binding protein 1
**IHC**	immunohistochemistry
**IL-10**	interleukin-10
**IL-1α**	interleukin-1 alpha
**IL-1β**	interleukin-1 beta
**IL-1Ra**	interleukin-1 receptor antagonist
**IL-2Ra**	interleukin-2 receptor antagonist
**IUA**	intrauterine adhesion
**KGF**	keratinocyte growth factor
**KGF-R**	keratocyte growth factor receptor
**LAP**	Lithium phenyl (2,4,6,-trimethylbenzoyl) phosphinate
**LPS**	lipopolysaccharide
**LRIG1**	leucine-rich repeats and immunoglubin-like domains 1
**LV**	left ventricular
**LVEF**	left ventricular ejection fraction
**MA**	methacrylic anhydride
**MBAA**	methylene bis acrylamide
**MEFs**	mouse embryonic fibroblasts
**MES**	2-(N-morpholino) ethane sulfonic
**MI**	myocardial infarction
**MIP-1α**	macrophage inflammatory protein-1 alpha
**MMP**	matrix metalloproteinase
**MMP-8**	matrix metallopeptidase 8
**MSCs**	mesenchymal stem cells
**NHS**	N-hydroxy sulfosuccinimide
**NICE**	National Institute for Health and Care Excellence
**NO**	nitrogen oxide
**NPs**	nanoparticles
**OA**	Osteoarthritis
**OECs**	outgrowth endothelial cells
**P407**	Poloxamer 407
**PAM**	polyacrylamide
**PCL**	polycaprolactone
**PDGF**	platelet-derived growth factor
**PECAM-1**	platelet-endothelial cell adhesion molecule-1
**PEDF**	pigment epithelium-derived factor
**PEGDA**	Polyethylene glycol diacrylate
**PF4**	platelet factor 4
**PGE2**	prostaglandin E2
**PGI**_**2**_	prostacyclin
**PlGF**	placenta growth factor
**PMSCs**	placenta-derived mesenchymal stem cells
**PVA**	Poly(vinyl alcohol)
**REDV**	Arg-Glu-Asp-Val peptide
**RGD**	Arginylglycylaspartic acid
**RM**	regenerative medicine
**RT**	room temperature
**SAM**	solubilized amnion
**sGAG**	sulfated glycosaminoglycan
**SLPI**	secretory leukocyte proteinase inhibitor
**SMCs**	smooth muscle cells
**TE**	tissue engineering
**TECs**	tissue-engineered constructs
**TEMED**	tetramethylethylenediamine
**TGF-α**	transforming growth factor-alpha
**TGF-β**	transforming growth factor-beta
**TIMP**	tissue inhibitors of metalloproteinase
**TNF-α**	tumor necrosis factor-alpha
**VEGF**	vascular endothelial growth factor
**VLAM**	viable lyophilized AM
**vWF**	von Willebrand factor
**WAP**	Whey acidic peptide

## References

[B1] Abaci A. Guvendiren M. (2020). Designing decellularized extracellular matrix‐based bioinks for 3D bioprinting. Adv. Healthc. Mater. 9 (24), 2000734. 10.1002/adhm.202000734 32691980

[B2] Abbasi-Kangevari M. Ghamari S.-H. Safaeinejad F. Bahrami S. Niknejad H. (2019). Potential therapeutic features of human amniotic mesenchymal stem cells in multiple sclerosis: immunomodulation, inflammation suppression, angiogenesis promotion, oxidative stress inhibition, neurogenesis induction, MMPs regulation, and remyelination stimulation. Front. Immunol. 10, 238. 10.3389/fimmu.2019.00238 30842772 PMC6391358

[B3] Abdo R. (2016). Treatment of diabetic foot ulcers with dehydrated amniotic membrane allograft: a prospective case series. J. Wound Care 25 (7), S4–S9. 10.12968/jowc.2016.25.Sup7.S4 29027851

[B4] Adipurnama I. Yang M.-C. Ciach T. Butruk-Raszeja B. (2017). Surface modification and endothelialization of polyurethane for vascular tissue engineering applications: a review. Biomaterials Sci. 5 (1), 22–37. 10.1039/C6BM00618C 27942617

[B5] Ajaykumar A. P. Mathew A. Chandni A. P. Varma S. R. Jayaraj K. N. Sabira O. (2023). Green synthesis of silver nanoparticles using the leaf extract of the medicinal plant, uvaria narum and its antibacterial, antiangiogenic, anticancer and catalytic properties. Antibiotics 12 (3), 564. 10.3390/antibiotics12030564 36978431 PMC10044571

[B6] Andrewartha N. Yeoh G. (2019). Human amnion epithelial cell therapy for chronic liver disease. Stem Cells Int. 2019, 1–10. 10.1155/2019/8106482 PMC6702811 31485235

[B7] Arki M. K. Moeinabadi-Bidgoli K. Hossein-Khannazer N. Gramignoli R. Najimi M. Vosough M. (2023). Amniotic membrane and its derivatives: novel therapeutic modalities in liver disorders. Cells 12 (16), 2114. 10.3390/cells12162114 37626924 PMC10453134

[B8] Arrizabalaga J. H. Nollert M. U. (2018). Human amniotic membrane: a versatile scaffold for tissue engineering. ACS Biomaterials Sci. Eng. 4 (7), 2226–2236. 10.1021/acsbiomaterials.8b00015 33435098

[B9] Babajani A. Manzari-Tavakoli A. Jamshidi E. Tarasi R. Niknejad H. (2022b). Anti-cancer effects of human placenta-derived amniotic epithelial stem cells loaded with paclitaxel on cancer cells. Sci. Rep. 12 (1), 18148. 10.1038/s41598-022-22562-w 36307463 PMC9616866

[B10] Babajani A. Moeinabadi-Bidgoli K. Niknejad F. Rismanchi H. Shafiee S. Shariatzadeh S. (2022a). Human placenta-derived amniotic epithelial cells as a new therapeutic hope for COVID-19-associated acute respiratory distress syndrome (ARDS) and systemic inflammation. Stem Cell Res. Ther. 13 (1), 126–222. 10.1186/s13287-022-02794-3 35337387 PMC8949831

[B11] Bakhshandeh H. Atyabi F. Soleimani M. Taherzadeh E. S. Shahhoseini S. Cohan R. A. (2021). Biocompatibility improvement of artificial cornea using chitosan-dextran nanoparticles containing bioactive macromolecules obtained from human amniotic membrane. Int. J. Biol. Macromol. 169, 492–499. 10.1016/j.ijbiomac.2020.12.125 33358948

[B12] Bakhtiar H. Ashoori A. Rajabi S. Pezeshki‐Modaress M. Ayati A. Mousavi M. R. (2022). Human amniotic membrane extracellular matrix scaffold for dental pulp regeneration *in vitro* and *in vivo* . Int. Endod. J. 55 (4), 374–390. 10.1111/iej.13675 34923640

[B13] Bakhtiar H. Mousavi M. R. Rajabi S. Pezeshki-Modaress M. Ayati A. Ashoori A. (2023). Fabrication and characterization of a novel injectable human amniotic membrane hydrogel for dentin-pulp complex regeneration. Dent. Mater. 39 (8), 718–728. 10.1016/j.dental.2023.06.008 37393152

[B14] Baradaran Rafii A. R. Aghayan H.-R. Arjmand B. Javadi M.-A. (2007). Amniotic membrane transplantation.

[B15] Barboni B. Russo V. Curini V. Mauro A. Martelli A. Muttini A. (2012). Achilles tendon regeneration can be improved by amniotic epithelial cell allotransplantation. Cell Transplant. 21 (11), 2377–2395. 10.3727/096368912X638892 22507232

[B16] Barr S. M. (2014). Dehydrated amniotic membrane allograft for treatment of chronic leg ulcers in patients with multiple comorbidities: a case series. J. Am. Coll. Clin. Wound Specialists 6 (3), 38–45. 10.1016/j.jccw.2016.01.002 PMC4828514 27104144

[B17] Bhattacharjee M. Escobar Ivirico J. L. Kan H.-M. Shah S. Otsuka T. Bordett R. (2022). Injectable amnion hydrogel-mediated delivery of adipose-derived stem cells for osteoarthritis treatment. Proc. Natl. Acad. Sci. 119 (4), e2120968119. 10.1073/pnas.2120968119 35046053 PMC8794776

[B18] Bhattacharjee M. Ivirico J. L. E. Kan H.-M. Bordett R. Pandey R. Otsuka T. (2020). Preparation and characterization of amnion hydrogel and its synergistic effect with adipose derived stem cells towards IL1β activated chondrocytes. Sci. Rep. 10 (1), 18751. 10.1038/s41598-020-75921-w 33127964 PMC7603317

[B19] Bianchi C. Cazzell S. Vayser D. Reyzelman A. M. Dosluoglu H. Tovmassian G. (2018). A multicentre randomised controlled trial evaluating the efficacy of dehydrated human amnion/chorion membrane (EpiFix^®^) allograft for the treatment of venous leg ulcers. Int. wound J. 15 (1), 114–122. 10.1111/iwj.12843 29024419 PMC7949978

[B20] Bianchi C. Tettelbach W. Istwan N. Hubbs B. Kot K. Harris S. (2019). Variations in study outcomes relative to intention‐to‐treat and per‐protocol data analysis techniques in the evaluation of efficacy for treatment of venous leg ulcers with dehydrated human amnion/chorion membrane allograft. Int. Wound J. 16 (3), 761–767. 10.1111/iwj.13094 30864259 PMC6850648

[B21] Biniazan F. Rajaei F. Darabi S. Babajani A. Mashayekhi M. Vousooghi N. (2022). Effects of placenta-derived human amniotic epithelial cells on the wound healing process and TGF-β induced scar formation in murine ischemic-reperfusion injury model. Stem Cell Rev. Rep. 18 (6), 2045–2058. 10.1007/s12015-022-10355-7 35303271

[B22] Blume G. G. Machado-Júnior P. A. B. Bertinato G. P. Simeoni R. B. Francisco J. C. Guarita-Souza L. C. (2021). Tissue-engineered amniotic membrane in the treatment of myocardial infarction: a systematic review of experimental studies. Am. J. Cardiovasc. Dis. 11 (1), 1–11. 33815914 PMC8012283

[B23] Bonvallet P. P. Damaraju S. M. Modi H. N. Stefanelli V. L. Lin Q. Saini S. (2022). Biophysical characterization of a novel tri-layer placental allograft membrane. Adv. Wound Care 11 (2), 43–55. 10.1089/wound.2020.1315 PMC9831246 33975444

[B24] Boyar V. Galiczewski C. (2018). Efficacy of dehydrated human amniotic membrane allograft for the treatment of severe extravasation injuries in preterm neonates. Wounds a Compend. Clin. Res. Pract. 30 (8), 224–228. 30212365

[B25] Chang B. Ahuja N. Ma C. Liu X. (2017). Injectable scaffolds: preparation and application in dental and craniofacial regeneration. Mater. Sci. Eng. R Rep. 111, 1–26. 10.1016/j.mser.2016.11.001 28649171 PMC5478172

[B26] Chen J. Wang M.-W. Xu J.-J. Wu X.-Y. Yao J. (2020). Gelatin methacryloyl hydrogel eye pad loaded with amniotic extract prevents symblepharon in rabbit eyes. Eur. Rev. Med. Pharmacol. Sci. 24 (19), 10134–10142. 10.26355/eurrev_202010_23233 33090421

[B27] Chen L. Ye J. Gao C. Deng F. Liu W. Zhang Q. (2023). Design and fabrication of gelatin-based hydrogel loaded with modified amniotic extracellular matrix for enhanced wound healing. Heliyon 9 (10), e20521. 10.1016/j.heliyon.2023.e20521 37790967 PMC10543223

[B28] Chen P. Lu M. Wang T. Dian D. Zhong Y. Aleahmad M. (2021). Human amniotic membrane as a delivery vehicle for stem cell-based therapies. Life Sci. 272, 119157. 10.1016/j.lfs.2021.119157 33524418

[B29] Chen X. Sun J. Li X. Mao L. Zhou Y. Cui L. (2018). Antifibrotic effects of decellularized and lyophilized human amniotic membrane transplant on the formation of intrauterine adhesion. Exp. Clin. Transplant. Official J. Middle East Soc. Organ Transplant. 17 (2), 236–242. 10.6002/ect.2017.0284 30251940

[B30] Cheng C. Peng X. Qi H. Wang X. Yu X. Wang Y. (2021). A promising potential candidate for vascular replacement materials with anti-inflammatory action, good hemocompatibility and endotheliocyte-cytocompatibility: phytic acid-fixed amniotic membrane. Biomed. Mater. 16 (6), 065009. 10.1088/1748-605X/ac246d 34492639

[B31] Chmait R. H. Kontopoulos E. V. Chon A. H. Korst L. M. Llanes A. Quintero R. A. (2017). Amniopatch treatment of iatrogenic preterm premature rupture of membranes (iPPROM) after fetoscopic laser surgery for twin–twin transfusion syndrome. J. Maternal-Fetal Neonatal Med. 30 (11), 1349–1354. 10.1080/14767058.2016.1214123 27686840

[B32] Choi C. M. Jeon H. S. (2022). Clinical outcomes of in-office sutureless amniotic membrane transplantation in persistent epithelial defect. Korean J. Ophthalmol. KJO. 36 (2), 87–96. 10.3341/kjo.2021.0095 34823345 PMC9013553

[B33] Chopra A. Thomas B. S. (2013). Amniotic membrane: a novel material for regeneration and repair. J. Biomim. Biomater. Tissue Eng. 18 (1), 1–8.

[B34] Choudhury D. Tun H. W. Wang T. Naing M. W. (2018). Organ-derived decellularized extracellular matrix: a game changer for bioink manufacturing? Trends Biotechnol. 36 (8), 787–805. 10.1016/j.tibtech.2018.03.003 29678431

[B35] Chun B. Y. Kim H. K. Shin J. P. (2013). Dried human amniotic membrane does not alleviate inflammation and fibrosis in experimental strabismus surgery. J. Ophthalmol. 2013, 1–6. 10.1155/2013/369126 PMC3705876 23864935

[B36] Comperat L. Chagot L. Massot S. Stachowicz M. L. Dusserre N. Médina C. (2023). Harnessing human placental membrane‐derived bioinks: characterization and applications in bioprinting and vasculogenesis. Adv. Healthc. Mater., 2303370. 10.1002/adhm.202303370 PMC11469061 37942849

[B37] Cornwell K. G. Landsman A. James K. S. (2009). Extracellular matrix biomaterials for soft tissue repair. Clin. podiatric Med. Surg. 26 (4), 507–523. 10.1016/j.cpm.2009.08.001 19778685

[B38] Cross M. J. Claesson-Welsh L. (2001). FGF and VEGF function in angiogenesis: signalling pathways, biological responses and therapeutic inhibition. Trends Pharmacol. Sci. 22 (4), 201–207. 10.1016/S0165-6147(00)01676-X 11282421

[B39] Cunningham B. W. Seiber B. Riggleman J. R. Van Horn M. R. Bhat A. (2019). An investigational study of a dual‐layer, chorion‐free amnion patch as a protective barrier following lumbar laminectomy in a sheep model. J. Tissue Eng. Regen. Med. 13 (9), 1664–1671. 10.1002/term.2920 31243876

[B40] Cunningham F. G. Leveno K. J. Bloom S. L. Spong C. Y. Dashe J. S. Hoffman B. L. (2014). Williams obstetrics. New York: McGraw-Hill Medical.

[B41] Dadkhah Tehrani F. Firouzeh A. Shabani I. Shabani A. (2021). A review on modifications of amniotic membrane for biomedical applications. Front. Bioeng. Biotechnol. 8, 606982. 10.3389/fbioe.2020.606982 33520961 PMC7839407

[B42] Davis J. (1910). Skin transplantation with a review of 550 cases at the johns hopkins hospital. Johns Hopkins Med. J. 15 (307), 307–398.

[B43] Deihim T. Yazdanpanah G. Niknejad H. (2016). Different light transmittance of placental and reflected regions of human amniotic membrane that could be crucial for corneal tissue engineering. Cornea 35 (7), 997–1003. 10.1097/ICO.0000000000000867 27149533

[B44] de la Torre P. Paris J. L. Fernández-de la Torre M. Vallet-Regí M. Flores A. I. (2021). Endostatin genetically engineered placental mesenchymal stromal cells carrying doxorubicin-loaded mesoporous silica nanoparticles for combined chemo-and antiangiogenic therapy. Pharmaceutics 13 (2), 244. 10.3390/pharmaceutics13020244 33578733 PMC7916487

[B45] De Santis M. M. Alsafadi H. N. Tas S. Bölükbas D. A. Prithiviraj S. Da Silva I. A. (2021). Extracellular‐matrix‐reinforced bioinks for 3D bioprinting human tissue. Adv. Mater. 33 (3), 2005476. 10.1002/adma.202005476 PMC11469085 33300242

[B46] Deus I. A. Santos S. C. Custódio C. A. Mano J. F. (2022). Designing highly customizable human based platforms for cell culture using proteins from the amniotic membrane. Biomater. Adv. 134, 112574. 10.1016/j.msec.2021.112574 35525741

[B47] Doucette M. Payne K. M. Lough W. Beck A. Wayment K. Huffman J. (2022). Early advanced therapy for diabetic foot ulcers in high amputation risk veterans: a cohort study. Int. J. Low. Extrem. Wounds 21 (2), 111–119. 10.1177/1534734620928151 32567415 PMC7752820

[B48] Elkhenany H. El-Derby A. Abd Elkodous M. Salah R. A. Lotfy A. El-Badri N. (2022). Applications of the amniotic membrane in tissue engineering and regeneration: the hundred-year challenge. Stem Cell Res. Ther. 13 (1), 8–19. 10.1186/s13287-021-02684-0 35012669 PMC8744057

[B49] Farhadihosseinabadi B. Farahani M. Tayebi T. Jafari A. Biniazan F. Modaresifar K. (2018). Amniotic membrane and its epithelial and mesenchymal stem cells as an appropriate source for skin tissue engineering and regenerative medicine. Artif. cells, nanomedicine, Biotechnol. 46 (2), 431–440. 10.1080/21691401.2018.1458730 29687742

[B50] Favaron P. O. Carvalho R. Borghesi J. Anunciação A. R.Ad Miglino M. A. (2015). The amniotic membrane: development and potential applications–a review. Reproduction Domest. animals 50 (6), 881–892. 10.1111/rda.12633 26510939

[B51] Fénelon M. Catros S. Meyer C. Fricain J.-C. Obert L. Auber F. (2021). Applications of human amniotic membrane for tissue engineering. Membranes 11 (6), 387. 10.3390/membranes11060387 34070582 PMC8227127

[B52] Fenelon M. Galvez P. Kalbermatten D. Scolozzi P. Madduri S. (2023). Emerging strategies for the biofabrication of multilayer composite amniotic membranes for biomedical applications. Int. J. Mol. Sci. 24 (19), 14424. 10.3390/ijms241914424 37833872 PMC10572287

[B53] Fetterolf D. E. Snyder R. J. (2012). Scientific and clinical support for the use of dehydrated amniotic membrane in wound management. Wounds: a compendium of clinical research and practice. Wounds 24 (10), 299–307. PMID: 25876055. 25876055

[B54] Fitriani N. Wilar G. Narsa A. C. Mohammed A. F. Wathoni N. (2023). Application of amniotic membrane in skin regeneration. Pharmaceutics 15 (3), 748. 10.3390/pharmaceutics15030748 36986608 PMC10053812

[B55] Fu Q. Ohnishi S. Sakamoto N. (2018). Conditioned medium from human amnion-derived mesenchymal stem cells regulates activation of primary hepatic stellate cells. Stem cells Int. 2018, 1–11. 10.1155/2018/4898152 PMC6196790 30402110

[B56] Fukuda K. Chikama T.-I. Nakamura M. Nishida T. (1999). Differential distribution of subchains of the basement membrane components type IV collagen and laminin among the amniotic membrane, cornea, and conjunctiva. Cornea 18 (1), 73–79. 10.1097/00003226-199901000-00013 9894941

[B57] Gaharwar A. K. Singh I. Khademhosseini A. (2020). Engineered biomaterials for *in situ* tissue regeneration. Nat. Rev. Mater. 5 (9), 686–705. 10.1038/s41578-020-0209-x

[B58] Garrido M. Escobar C. Zamora C. Rejas C. Varas J. Córdova C. (2018). Transplantation of human amniotic membrane over the liver surface reduces hepatic fibrosis in a cholestatic model in young rats. Stem Cells Int. 2018, 1–9. 10.1155/2018/6169546 PMC5845510 29535774

[B59] Ghazani M. A. Soltani M. Jalali P. Hassannejad R. (2022). A novel numerical and artificial intelligence based approach to study anti-angiogenic drugs: endostatin. Appl. Math. Model. 105, 258–283. 10.1016/j.apm.2021.12.033

[B60] Gholipourmalekabadi M. Farhadihosseinabadi B. Faraji M. Nourani M. R. (2020). How preparation and preservation procedures affect the properties of amniotic membrane? How safe are the procedures? Burns 46 (6), 1254–1271. 10.1016/j.burns.2019.07.005 31445711

[B61] Gicquel J.-J. Dua H. S. Brodie A. Mohammed I. Suleman H. Lazutina E. (2009). Epidermal growth factor variations in amniotic membrane used for *ex vivo* tissue constructs. Tissue Eng. Part A 15 (8), 1919–1927. 10.1089/ten.tea.2008.0432 19196134

[B62] Gonçalves J. Tannuri A. Coelho M. Bendit I. Tannuri U. (2014). Dynamic expression of desmin, α-SMA and TGF-β1 during hepatic fibrogenesis induced by selective bile duct ligation in young rats. Braz. J. Med. Biol. Res. 47, 850–857. 10.1590/1414-431X20143679 25140817 PMC4181220

[B63] Grewal D. S. Mahmoud T. H. (2016). Dehydrated allogenic human amniotic membrane graft for conjunctival surface reconstruction following removal of exposed scleral buckle. Ophthalmic Surg. Lasers Imaging Retina 47 (10), 948–951. 10.3928/23258160-20161004-08 27759861

[B64] Guaní-Guerra E. Santos-Mendoza T. Lugo-Reyes S. O. Terán L. M. (2010). Antimicrobial peptides: general overview and clinical implications in human health and disease. Clin. Immunol. 135 (1), 1–11. 10.1016/j.clim.2009.12.004 20116332

[B65] Haghshenas M. Tavana S. Zand E. Montazeri L. Fathi R. (2022). Mouse ovarian follicle growth in an amniotic membrane-based hydrogel. J. Biomaterials Appl. 37 (3), 563–574. 10.1177/08853282221094193 35451867

[B66] Hao Y. Ma D. H.-K. Hwang D. G. Kim W.-S. Zhang F. (2000). Identification of antiangiogenic and antiinflammatory proteins in human amniotic membrane. Cornea 19 (3), 348–352. 10.1097/00003226-200005000-00018 10832697

[B67] Hashimoto H. Olson E. N. Bassel-Duby R. (2018). Therapeutic approaches for cardiac regeneration and repair. Nat. Rev. Cardiol. 15 (10), 585–600. 10.1038/s41569-018-0036-6 29872165 PMC6241533

[B68] Heidari F. Saadatmand M. Simorgh S. (2023). Directly coaxial bioprinting of 3D vascularized tissue using novel bioink based on decellularized human amniotic membrane. Int. J. Biol. Macromol. 253, 127041. 10.1016/j.ijbiomac.2023.127041 37742904

[B69] Heinegård D. Saxne T. (2011). The role of the cartilage matrix in osteoarthritis. Nat. Rev. Rheumatol. 7 (1), 50–56. 10.1038/nrrheum.2010.198 21119607

[B70] Henry J. J. Delrosario L. Fang J. Wong S. Y. Fang Q. Sievers R. (2020). Development of injectable amniotic membrane matrix for postmyocardial infarction tissue repair. Adv. Healthc. Mater. 9 (2), 1900544. 10.1002/adhm.201900544 PMC6986802 31778043

[B71] Hinderland M. D. Alan N. (2012). Revisional tarsal tunnel decompression with Allowrap® DS. Allow. Clin. Rep. Ser., 1–4.

[B72] Hofmann N. Salz A.-K. Kleinhoff K. Möhle N. Börgel M. Diedenhofen N. (2021). AmnioClip-plus as sutureless alternative to amniotic membrane transplantation to improve healing of ocular surface disorders. Transplantology 2 (4), 425–432. 10.3390/transplantology2040040

[B73] Honjo K.-I. Yamamoto T. Adachi T. Amemiya T. Mazda O. Kanamura N. (2015). Evaluation of a dental pulp-derived cell sheet cultured on amniotic membrane substrate. Bio-Medical Mater. Eng. 25 (2), 203–212. 10.3233/BME-151270 25813958

[B74] Hooker A. B. Lemmers M. Thurkow A. L. Heymans M. W. Opmeer B. C. Brölmann H. A. (2014). Systematic review and meta-analysis of intrauterine adhesions after miscarriage: prevalence, risk factors and long-term reproductive outcome. Hum. Reprod. update 20 (2), 262–278. 10.1093/humupd/dmt045 24082042

[B75] Hori J. Wang M. Kamiya K. Takahashi H. Sakuragawa N. (2006). Immunological characteristics of amniotic epithelium. Cornea 25, S53–S58. 10.1097/01.ico.0000247214.31757.5c 17001194

[B76] Horn A. Saller J. Cuttica D. Neufeld S. (2019). Review of use of amniotic membrane allograft in total ankle replacements. Foot Ankle Orthop. 4 (4), 2473011419S0022. 10.1177/2473011419S00222

[B77] Hossain L. Siddika A. Adnan M. Diba F. Hasan Z. Asaduzzaman S. (2019). Human amniotic membrane and its anti-cancer mechanism: a good hope for cancer therapy. SN Compr. Clin. Med. 1 (7), 487–495. 10.1007/s42399-019-00090-5

[B78] Hossain M. L. Rahman M. A. Siddika A. Adnan M. Rahman H. Diba F. (2020). Burn and wound healing using radiation sterilized human amniotic membrane and centella asiatica derived gel: a review. Regen. Eng. Transl. Med. 6, 347–357. 10.1007/s40883-019-00122-5

[B79] Hu Z. Luo Y. Ni R. Hu Y. Yang F. Du T. (2023). Biological importance of human amniotic membrane in tissue engineering and regenerative medicine. Mater. Today Bio 22, 100790. 10.1016/j.mtbio.2023.100790 PMC10498009 37711653

[B80] Huang Y. Bai L. Yang Y. Yin Z. Guo B. (2022). Biodegradable gelatin/silver nanoparticle composite cryogel with excellent antibacterial and antibiofilm activity and hemostasis for Pseudomonas aeruginosa-infected burn wound healing. J. Colloid Interface Sci. 608, 2278–2289. 10.1016/j.jcis.2021.10.131 34774324

[B81] Hussin I. Pingguan-Murphy B. Osman S. (2011). “The fabrication of human amniotic membrane based hydrogel for cartilage tissue engineering applications: a preliminary study,” in 5th Kuala Lumpur International Conference on Biomedical Engineering 2011: (BIOMED 2011), Kuala Lumpur, Malaysia, 20-23 June 2011. (Springer). 10.1007/978-3-642-21729-6_205

[B82] Islam M. Karmakar P. C. Arifuzzaman M. Karim N. Akhtar N. Asaduzzaman S. (2023). Human amniotic membrane and titanium dioxide nanoparticle derived gel for burn wound healing in a rat model. Regen. Eng. Transl. Med. 9 (2), 249–262. 10.1007/s40883-022-00280-z

[B83] Jafari A. Niknejad H. Rezaei-Tavirani M. Sarrami-Forooshani R. Gilanchi S. Jafari Z. (2023). Antiproliferative and apoptotic effects of conditioned medium released from human amniotic epithelial stem cells on breast and cervical cancer cells. Int. J. Immunopathol. Pharmacol. 37, 039463202211507. 10.1177/03946320221150712 PMC9841833 36638388

[B84] Jafari A. Rezaei-Tavirani M. Farhadihosseinabadi B. Zali H. Niknejad H. (2021). Human amniotic mesenchymal stem cells to promote/suppress cancer: two sides of the same coin. Stem Cell Res. Ther. 12, 126–211. 10.1186/s13287-021-02196-x 33579346 PMC7881457

[B85] Jahanafrooz Z. Bakhshandeh B. Behnam Abdollahi S. Seyedjafari E. (2023). Human amniotic membrane as a multifunctional biomaterial: recent advances and applications. J. Biomaterials Appl. 37 (8), 1341–1354. 10.1177/08853282221137609 36331116

[B86] Jhumi I. J. Arafat T.-A. Karmakar P. C. Arifuzzaman M. Hossain M. S. Akhtar N. (2023). Silver nanoparticle incorporated human amniotic membrane gel accelerates second-degree burn wound healing in wister rat. Evidence-Based Complementary Altern. Med. 2023, 1–15. 10.1155/2023/9808556 PMC10121346 37089708

[B87] John T. (2003). Human amniotic membrane transplantation: past, present, and future. Ophthalmol. Clin. N. Am. 16 (1), 43–65. vi. 10.1016/s0896-1549(02)00110-4 12683248

[B88] Johnson T. D. Lin S. Y. Christman K. L. (2011). Tailoring material properties of a nanofibrous extracellular matrix derived hydrogel. Nanotechnology 22 (49), 494015. 10.1088/0957-4484/22/49/494015 22101810 PMC3280097

[B89] Ju Y. Tang Z. Dai X. Gao H. Zhang J. Liu Y. (2022). Protection against light‐induced retinal degeneration via dual anti‐inflammatory and anti‐angiogenic functions of thrombospondin‐1. Br. J. Pharmacol. 179 (9), 1938–1961. 10.1111/bph.15303 33125704

[B90] Kafili G. Kabir H. Jalali Kandeloos A. Golafshan E. Ghasemi S. Mashayekhan S. (2023c). Recent advances in soluble decellularized extracellular matrix for heart tissue engineering and organ modeling. J. Biomaterials Appl. 38 (5), 577–604. 10.1177/08853282231207216 PMC10676626 38006224

[B91] Kafili G. Tamjid E. Niknejad H. Simchi A. (2022). Development of injectable hydrogels based on human amniotic membrane and polyethyleneglycol-modified nanosilicates for tissue engineering applications. Eur. Polym. J. 179, 111566. 10.1016/j.eurpolymj.2022.111566

[B92] Kafili G. Tamjid E. niknejad H. Simchi A. (2023a). Effect of pepsin digestion time on the properties of temperature sensitive human amniotic membrane derived hydrogel. Modares J. Biotechnol. 13 (3), 113–131. Available at: http://biot.modares.ac.ir/article-22-51635-fa.html.

[B93] Kafili G. Tamjid E. Niknejad H. Simchi A. (2023b). Development of printable nanoengineered composite hydrogels based on human amniotic membrane for wound healing application. J. Mater. Sci. 58, 12351–12372. 10.1007/s10853-023-08783-y

[B94] Kapasi K. Albert S. Yie S. M. Zavazava N. Librach C. (2000). HLA‐G has a concentration‐dependent effect on the generation of an allo‐CTL response. Immunology 101 (2), 191–200. 10.1046/j.1365-2567.2000.00109.x 11012772 PMC2327080

[B95] Khalatbary A. R. Omraninava M. Nasiry D. Akbari M. Taghiloo S. Poorhassan M. (2023). Exosomes derived from human adipose mesenchymal stem cells loaded bioengineered three-dimensional amniotic membrane-scaffold-accelerated diabetic wound healing. Archives Dermatological Res. 315 (10), 2853–2870. 10.1007/s00403-023-02709-z 37644140

[B96] Kim B. S. Das S. Jang J. Cho D.-W. (2020). Decellularized extracellular matrix-based bioinks for engineering tissue-and organ-specific microenvironments. Chem. Rev. 120 (19), 10608–10661. 10.1021/acs.chemrev.9b00808 32786425

[B97] Kim B. S. Kim H. Gao G. Jang J. Cho D.-W. (2017). Decellularized extracellular matrix: a step towards the next generation source for bioink manufacturing. Biofabrication 9 (3), 034104. 10.1088/1758-5090/aa7e98 28691696

[B98] Kim G. H. Uriel N. Burkhoff D. (2018). Reverse remodelling and myocardial recovery in heart failure. Nat. Rev. Cardiol. 15 (2), 83–96. 10.1038/nrcardio.2017.139 28933783

[B99] Kim H. S. Cho J. H. Park H. W. Yoon H. Kim M. S. Kim S. C. (2002). Endotoxin-neutralizing antimicrobial proteins of the human placenta. J. Immunol. 168 (5), 2356–2364. 10.4049/jimmunol.168.5.2356 11859126

[B100] Kim J. S. Kim J. C. Na B. K. Jeong J. M. Song C. Y. (2000). Amniotic membrane patching promotes healing and inhibits proteinase activity on wound healing following acute corneal alkali burn. Exp. eye Res. 70 (3), 329–337. 10.1006/exer.1999.0794 10712819

[B101] Kim J.-Y. Lee S. Y. Park S.-C. Shin S. Y. Choi S. J. Park Y. (2007). Purification and antimicrobial activity studies of the N-terminal fragment of ubiquitin from human amniotic fluid. Biochimica Biophysica Acta (BBA)-Proteins Proteomics 1774 (9), 1221–1226. 10.1016/j.bbapap.2007.06.013 17669700

[B102] Kim S. G. Zhou J. Solomon C. Zheng Y. Suzuki T. Chen M. (2012). Effects of growth factors on dental stem/progenitor cells. Dent. Clin. N. Am. 56 (3), 563–575. 10.1016/j.cden.2012.05.001 22835538 PMC4112411

[B103] King A. Paltoo A. Kelly R. Sallenave J.-M. Bocking A. Challis J. (2007). Expression of natural antimicrobials by human placenta and fetal membranes. Placenta 28 (2-3), 161–169. 10.1016/j.placenta.2006.01.006 16513165

[B104] Kobayashi N. Kabuyama Y. Sasaki S. Kato K.-I. Homma Y. (2002). Suppression of corneal neovascularization by culture supernatant of human amniotic cells. Cornea 21 (1), 62–67. 10.1097/00003226-200201000-00014 11805510

[B105] Koelink P. J. Bloemendaal F. M. Li B. Westera L. Vogels E. W. van Roest M. (2020). Anti-TNF therapy in IBD exerts its therapeutic effect through macrophage IL-10 signalling. Gut 69 (6), 1053–1063. 10.1136/gutjnl-2019-318264 31506328 PMC7282553

[B106] Koizumi N. Inatomi T. Sotozono C. Fullwood N. J. Quantock A. J. Kinoshita S. (2000). Growth factor mRNA and protein in preserved human amniotic membrane. Curr. eye Res. 20 (3), 173–177. 10.1076/0271-3683(200003)2031-9FT173 10694891

[B107] Kotomin I. Valtink M. Hofmann K. Frenzel A. Morawietz H. Werner C. (2015). Sutureless fixation of amniotic membrane for therapy of ocular surface disorders. PLoS One 10 (5), e0125035. 10.1371/journal.pone.0125035 25955359 PMC4425509

[B108] Krafts K. P. (2010). Tissue repair: the hidden drama. Organogenesis 6 (4), 225–233. 10.4161/org.6.4.12555 21220961 PMC3055648

[B109] Kraitchman D. L. Tatsumi M. Gilson W. D. Ishimori T. Kedziorek D. Walczak P. (2005). Dynamic imaging of allogeneic mesenchymal stem cells trafficking to myocardial infarction. Circulation 112 (10), 1451–1461. 10.1161/CIRCULATIONAHA.105.537480 16129797 PMC1456731

[B110] Lacorzana J. (2020). Amniotic membrane, clinical applications and tissue engineering. Review of its ophthalmic use. Arch. Soc. Española Oftalmol. (English Ed). 95 (1), 15–23. 10.1016/j.oftale.2019.09.008 31784120

[B111] Lee J. Hong J. Kim W. Kim G. H. (2020). Bone-derived dECM/alginate bioink for fabricating a 3D cell-laden mesh structure for bone tissue engineering. Carbohydr. Polym. 250, 116914. 10.1016/j.carbpol.2020.116914 33049834

[B112] Lee J. Y. Kim H. Ha D. H. Shin J. C. Kim A. Ko H. S. (2018). Amnion‐analogous medical device for fetal membrane healing: a preclinical long‐term study. Adv. Healthc. Mater. 7 (18), 1800673. 10.1002/adhm.201800673 30133182

[B113] Lei X. Wu Y. Peng X. Zhao Y. Zhou X. Yu X. (2020). Research on alginate-polyacrylamide enhanced amnion hydrogel, a potential vascular substitute material. Mater. Sci. Eng. C 115, 111145. 10.1016/j.msec.2020.111145 32600732

[B114] Li H. Niederkorn J. Y. Neelam S. Mayhew E. Word R. A. McCulley J. P. (2005). Immunosuppressive factors secreted by human amniotic epithelial cells. Investigative Ophthalmol. Vis. Sci. 46 (3), 900–907. 10.1167/iovs.04-0495 15728546

[B115] Li X. Li P. Wang C. Shang T. Han H. Tong Y. (2022). A thermo-sensitive and injectable hydrogel derived from a decellularized amniotic membrane to prevent intrauterine adhesion by accelerating endometrium regeneration. Biomaterials Sci. 10 (9), 2275–2286. 10.1039/D1BM01791H 35363229

[B116] Lintzeris D. Yarrow K. Johnson L. White A. Hampton A. Strickland A. (2015). Use of a dehydrated amniotic membrane allograft on lower extremity ulcers in patients with challenging wounds: a retrospective case series. Ostomy/wound Manag. 61 (10), 30–36. 26479124

[B117] Lipový B. Hladík M. Štourač P. Forostyak S. (2021). Case report: wound closure acceleration in a patient with toxic epidermal necrolysis using a lyophilised amniotic membrane. Front. Bioeng. Biotechnol. 9, 649317. 10.3389/fbioe.2021.649317 33937217 PMC8085411

[B118] Liu C. Bai J. Yu K. Liu G. Tian S. Tian D. (2019). Biological amnion prevents flexor tendon adhesion in zone II: a controlled, multicentre clinical trial. BioMed Res. Int. 2019, 1–9. 10.1155/2019/2354325 PMC6470416 31073521

[B119] Liu Q.-W. Ying Y.-M. Zhou J.-X. Zhang W.-J. Liu Z.-X. Jia B.-B. (2022). Human amniotic mesenchymal stem cells-derived IGFBP-3, DKK-3, and DKK-1 attenuate liver fibrosis through inhibiting hepatic stellate cell activation by blocking Wnt/β-catenin signaling pathway in mice. Stem Cell Res. Ther. 13 (1), 224–318. 10.1186/s13287-022-02906-z 35659360 PMC9166579

[B120] Liu Y. H. Vaghjiani V. Tee J. Y. To K. Cui P. Oh D. Y. (2012). Amniotic epithelial cells from the human placenta potently suppress a mouse model of multiple sclerosis. PloS one 7 (4), e35758. 10.1371/journal.pone.0035758 22563398 PMC3338525

[B121] Lohajaroensub R. Sawangmake C. Rodkhum C. Tuntivanich N. (2022). Expression of antimicrobial peptide genes in the canine amniotic membrane. Veterinary Sci. 9 (5), 200. 10.3390/vetsci9050200 PMC9146009 35622728

[B122] Lokhande G. Carrow J. K. Thakur T. Xavier J. R. Parani M. Bayless K. J. (2018). Nanoengineered injectable hydrogels for wound healing application. Acta biomater. 70, 35–47. 10.1016/j.actbio.2018.01.045 29425720 PMC7499308

[B123] Luo Y. Shen M. Feng P. Qiu H. Wu X. Yang L. (2021). Various administration forms of decellularized amniotic membrane extract towards improving corneal repair. J. Mater. Chem. B 9 (45), 9347–9357. 10.1039/D1TB01848E 34724021

[B124] Lynn A. Yannas I. Bonfield W. (2004). Antigenicity and immunogenicity of collagen. J. Biomed. Mater. Res. Part B Appl. Biomaterials 71 (2), 343–354. 10.1002/jbm.b.30096 15386396

[B125] Magatti M. Caruso M. De Munari S. Vertua E. De D. Manuelpillai U. (2015). Human amniotic membrane-derived mesenchymal and epithelial cells exert different effects on monocyte-derived dendritic cell differentiation and function. Cell Transplant. 24 (9), 1733–1752. 10.3727/096368914X684033 25259480

[B126] Magatti M. Masserdotti A. Bonassi Signoroni P. Vertua E. Stefani F. R. Silini A. R. (2020). B lymphocytes as targets of the immunomodulatory properties of human amniotic mesenchymal stromal cells. Front. Immunol. 11, 1156. 10.3389/fimmu.2020.01156 32582218 PMC7295987

[B127] Magatti M. Vertua E. Cargnoni A. Silini A. Parolini O. (2018). The immunomodulatory properties of amniotic cells: the two sides of the coin. Cell Transplant. 27 (1), 31–44. 10.1177/0963689717742819 29562786 PMC6434482

[B128] Magatti M. Vertua E. De Munari S. Caro M. Caruso M. Silini A. (2017). Human amnion favours tissue repair by inducing the M1‐to‐M2 switch and enhancing M2 macrophage features. J. tissue Eng. Regen. Med. 11 (10), 2895–2911. 10.1002/term.2193 27396853 PMC5697700

[B129] Mamede A. C. Carvalho M. Abrantes A. M. Laranjo M. Maia C. Botelho M. (2012). Amniotic membrane: from structure and functions to clinical applications. Cell tissue Res. 349, 447–458. 10.1007/s00441-012-1424-6 22592624

[B130] Manuelpillai U. Tchongue J. Lourensz D. Vaghjiani V. Samuel C. S. Liu A. (2010). Transplantation of human amnion epithelial cells reduces hepatic fibrosis in immunocompetent CCl4-treated mice. Cell Transplant. 19 (9), 1157–1168. 10.3727/096368910X504496 20447339

[B131] Mao Y. Hoffman T. Dhall S. Singal A. Sathyamoorthy M. Danilkovitch A. (2019). Endogenous viable cells in lyopreserved amnion retain differentiation potential and anti-fibrotic activity *in vitro* . Acta Biomater. 94, 330–339. 10.1016/j.actbio.2019.06.002 31176843

[B132] Mao Y. Hoffman T. Singh-Varma A. Duan-Arnold Y. Moorman M. Danilkovitch A. (2017). Antimicrobial peptides secreted from human cryopreserved viable amniotic membrane contribute to its antibacterial activity. Sci. Rep. 7 (1), 13722. 10.1038/s41598-017-13310-6 29057887 PMC5651856

[B133] Margiana R. Markov A. Zekiy A. O. Hamza M. U. Al-Dabbagh K. A. Al-Zubaidi S. H. (2022). Clinical application of mesenchymal stem cell in regenerative medicine: a narrative review. Stem Cell Res. Ther. 13 (1), 366–422. 10.1186/s13287-022-03054-0 35902958 PMC9330677

[B134] McQuilling J. P. Sanders M. Poland L. Sanders M. Basadonna G. Waldrop N. E. (2019). Dehydrated amnion/chorion improves Achilles tendon repair in a diabetic animal model. Wounds a Compend. Clin. Res. Pract. 31 (1), 19–25. PMC7989034 30372415

[B135] McQuilling J. P. Vines J. B. Kimmerling K. A. Mowry K. C. (2017b). Proteomic comparison of amnion and chorion and evaluation of the effects of processing on placental membranes. Wounds a Compend. Clin. Res. Pract. 29 (6), E36–E40. PMC8009308 28682294

[B136] McQuilling J. P. Vines J. B. Mowry K. C. (2017a). *In vitro* assessment of a novel, hypothermically stored amniotic membrane for use in a chronic wound environment. Int. wound J. 14 (6), 993–1005. 10.1111/iwj.12748 28370981 PMC7949938

[B137] Mohan R. Bajaj A. Gundappa M. (2017). Human amnion membrane: potential applications in oral and periodontal field. J. Int. Soc. Prev. Community Dent. 7 (1), 15. 10.4103/jispcd.JISPCD_359_16 28316944 PMC5343678

[B138] Moussa D. G. Aparicio C. (2019). Present and future of tissue engineering scaffolds for dentin‐pulp complex regeneration. J. tissue Eng. Regen. Med. 13 (1), 58–75. 10.1002/term.2769 30376696 PMC6338516

[B139] Munoz-Torres J. R. Martínez-González S. B. Lozano-Luján A. D. Martínez-Vázquez M. C. Velasco-Elizondo P. Garza-Veloz I. (2023). Biological properties and surgical applications of the human amniotic membrane. Front. Bioeng. Biotechnol. 10, 1067480. 10.3389/fbioe.2022.1067480 36698632 PMC9868191

[B140] Murphy M. B. Moncivais K. Caplan A. I. (2013). Mesenchymal stem cells: environmentally responsive therapeutics for regenerative medicine. Exp. Mol. Med. 45 (11), e54. 10.1038/emm.2013.94 24232253 PMC3849579

[B141] Murphy S. V. Skardal A. Nelson R. A. Jr Sunnon K. Reid T. Clouse C. (2020). Amnion membrane hydrogel and amnion membrane powder accelerate wound healing in a full thickness porcine skin wound model. Stem cells Transl. Med. 9 (1), 80–92. 10.1002/sctm.19-0101 31328435 PMC6954699

[B142] Murphy S. V. Skardal A. Song L. Sutton K. Haug R. Mack D. L. (2017). Solubilized amnion membrane hyaluronic acid hydrogel accelerates full-thickness wound healing. Stem cells Transl. Med. 6 (11), 2020–2032. 10.1002/sctm.17-0053 28941321 PMC6430059

[B143] Murri M. S. Moshirfar M. Birdsong O. C. Ronquillo Y. C. Ding Y. Hoopes P. C. (2018). Amniotic membrane extract and eye drops: a review of literature and clinical application. Clin. Ophthalmol. 12, 1105–1112. 10.2147/OPTH.S165553 29950805 PMC6012548

[B144] Nagaoka H. Nagaoka H. Walter R. Boushell L. W. Miguez P. A. Burton A. (2014). Characterization of genipin-modified dentin collagen. BioMed Res. Int. 2014, 1–7. 10.1155/2014/702821 PMC3984863 24795891

[B145] Nasiry D. Khalatbary A. R. Abdollahifar M.-A. Amini A. Bayat M. Noori A. (2021). Engraftment of bioengineered three-dimensional scaffold from human amniotic membrane-derived extracellular matrix accelerates ischemic diabetic wound healing. Archives Dermatological Res. 313 (7), 567–582. 10.1007/s00403-020-02137-3 32940766

[B146] Nasiry D. Khalatbary A. R. Abdollahifar M.-A. Bayat M. Amini A. Ashtiani M. K. (2022). SDF-1α loaded bioengineered human amniotic membrane-derived scaffold transplantation in combination with hyperbaric oxygen improved diabetic wound healing. J. Biosci. Bioeng. 133 (5), 489–501. 10.1016/j.jbiosc.2022.01.012 35248486

[B147] Nasiry D. Khalatbary A. R. Noori A. Abouhamzeh B. Jamalpoor Z. (2023). Accelerated wound healing using three-dimensional amniotic membrane scaffold in combination with adipose-derived stem cells in a diabetic rat model. Tissue Cell 82, 102098. 10.1016/j.tice.2023.102098 37121056

[B148] Naxer S. Horn M. Schittkowski M. (2018). Processed amniotic membrane for conjunctival reconstruction in complex strabismus surgery. Strabismus 26 (4), 191–197. 10.1080/09273972.2018.1502794 30130446

[B149] Nazanin M. Mahshad M. Mehdi B. Farzaneh C. (2022). Enhanced survival and accelerated perfusion of skin flap to recipient site following administration of human amniotic membrane in rat models. J. Plastic, Reconstr. Aesthetic Surg. 75 (11), 4321–4327. 10.1016/j.bjps.2022.08.028 36229314

[B150] Nejad A. R. Hamidieh A. A. Amirkhani M. A. Sisakht M. M. (2021). Update review on five top clinical applications of human amniotic membrane in regenerative medicine. Placenta 103, 104–119. 10.1016/j.placenta.2020.10.026 33120046

[B151] Newman J. H. Rich S. Abman S. H. Alexander J. H. Barnard J. Beck G. J. (2017). Enhancing insights into pulmonary vascular disease through a precision medicine approach. A joint NHLBI–Cardiovascular Medical Research and Education Fund Workshop Report. Am. J. Respir. Crit. care Med. 195 (12), 1661–1670. 10.1164/rccm.201701-0150WS 28430547 PMC5476915

[B152] Ni J. Abrahamson M. Zhang M. Fernandez M. A. Grubb A. Su J. (1997). Cystatin E is a novel human cysteine proteinase inhibitor with structural resemblance to family 2 cystatins. J. Biol. Chem. 272 (16), 10853–10858. 10.1074/jbc.272.16.10853 9099741

[B153] Niknejad H. Paeini-Vayghan G. Tehrani F. Khayat-Khoei M. Peirovi H. (2013). Side dependent effects of the human amnion on angiogenesis. Placenta 34 (4), 340–345. 10.1016/j.placenta.2013.02.001 23465536

[B154] Niknejad H. Peirovi H. Jorjani M. Ahmadiani A. Ghanavi J. Seifalian A. M. (2008). Properties of the amniotic membrane for potential use in tissue engineering. Eur. Cells Mater 15, 88–99. 10.22203/ecm.v015a07 18446690

[B155] Niknejad H. Yazdanpanah G. (2014). Opposing effect of amniotic membrane on angiogenesis originating from amniotic epithelial cells. J. Med. Hypotheses Ideas 8 (1), 39–41. 10.1016/j.jmhi.2013.08.002

[B156] Nilforoushzadeh M. A. Amirkhani M. A. Hamidieh A. A. Seifalian A. M. Sisakht M. M. (2019). Skin regenerative medicine advancements in the Islamic Republic of Iran: a concise review. Regen. Med. 14 (11), 1047–1056. 10.2217/rme-2018-0170 31718464

[B157] Nouri M. Ebrahimi M. Bagheri T. Fatemi M. J. Najafbeygi A. Araghi S. (2018). Healing effects of dried and acellular human amniotic membrane and mepitelas for coverage of skin graft donor areas; a randomized clinical trial. Bull. Emerg. Trauma 6 (3), 195–200. 10.29252/beat-060302 30090813 PMC6078477

[B158] Ohno M. Martinez-Hernandez A. Ohno N. Kefalides N. A. (1983). Isolation of laminin from human placental basement membranes: amnion, chorion and chorionic microvessels. Biochem. Biophysical Res. Commun. 112 (3), 1091–1098. 10.1016/0006-291X(83)91730-8 6847680

[B159] Parolini O. Alviano F. Bagnara G. P. Bilic G. Bühring H.-J. Evangelista M. (2008). Concise review: isolation and characterization of cells from human term placenta: outcome of the first international Workshop on Placenta Derived Stem Cells. Stem cells 26 (2), 300–311. 10.1634/stemcells.2007-0594 17975221

[B160] Parolini O. Soncini M. Evangelista M. Schmidt D. (2009). Amniotic membrane and amniotic fluid-derived cells: potential tools for regenerative medicine? Regen. Med. 4, 275–291. 10.2217/17460751.4.2.275 19317646

[B161] Peng X. Wang X. Cheng C. Zhou X. Gu Z. Li L. (2020). Bioinspired, artificial, small-diameter vascular grafts with selective and rapid endothelialization based on an amniotic membrane-derived hydrogel. ACS Biomaterials Sci. Eng. 6 (3), 1603–1613. 10.1021/acsbiomaterials.9b01493 33455393

[B162] Phinney D. G. Prockop D. J. (2007). Concise review: mesenchymal stem/multipotent stromal cells: the state of transdifferentiation and modes of tissue repair—current views. Stem cells 25 (11), 2896–2902. 10.1634/stemcells.2007-0637 17901396

[B163] Pober J. S. Sessa W. C. (2015). Inflammation and the blood microvascular system. Cold Spring Harb. Perspect. Biol. 7 (1), a016345. 10.1101/cshperspect.a016345 PMC4292166 25384307

[B164] Pogozhykh O. Hofmann N. Gryshkov O. von Kaisenberg C. Mueller M. Glasmacher B. (2020). Repeated freezing procedures preserve structural and functional properties of amniotic membrane for application in ophthalmology. Int. J. Mol. Sci. 21 (11), 4029. 10.3390/ijms21114029 32512889 PMC7312941

[B165] Poss K. D. (2010). Advances in understanding tissue regenerative capacity and mechanisms in animals. Nat. Rev. Genet. 11 (10), 710–722. 10.1038/nrg2879 20838411 PMC3069856

[B166] Pourali P. Yahyaei B. (2016). Biological production of silver nanoparticles by soil isolated bacteria and preliminary study of their cytotoxicity and cutaneous wound healing efficiency in rat. J. Trace Elem. Med. Biol. 34, 22–31. 10.1016/j.jtemb.2015.11.004 26854241

[B167] Price C. A. (2016). Mechanisms of fibroblast growth factor signaling in the ovarian follicle. J. Endocrinol. 228 (2), R31–R43. 10.1530/joe-15-0414 26542145

[B168] Radke D. Jia W. Sharma D. Fena K. Wang G. Goldman J. (2018). Tissue engineering at the blood‐contacting surface: a review of challenges and strategies in vascular graft development. Adv. Healthc. Mater. 7 (15), 1701461. 10.1002/adhm.201701461 PMC6105365 29732735

[B169] Rahman M. S. Islam R. Rana M. M. Spitzhorn L.-S. Rahman M. S. Adjaye J. (2019). Characterization of burn wound healing gel prepared from human amniotic membrane and Aloe vera extract. BMC Complementary Altern. Med. 19, 115–15. 10.1186/s12906-019-2525-5 PMC6547555 31159783

[B170] Rana M. M. Rahman M. S. Ullah M. A. Siddika A. Hossain M. L. Akhter M. S. (2020). Amnion and collagen-based blended hydrogel improves burn healing efficacy on a rat skin wound model in the presence of wound dressing biomembrane. Bio-Medical Mater. Eng. 31 (1), 1–17. 10.3233/BME-201076 32144968

[B171] R Mohan R. Ck Tovey J. Gupta R. Sharma A. Tandon A. (2011). Decorin biology, expression, function and therapy in the cornea. Curr. Mol. Med. 11 (2), 110–128. 10.2174/156652411794859241 21342131

[B172] Rosenblum B. I. (2016). A retrospective case series of a dehydrated amniotic membrane allograft for treatment of unresolved diabetic foot ulcers. J. Am. Podiatric Med. Assoc. 106 (5), 328–337. 10.7547/15-139 27439322

[B173] Ryzhuk V. Zeng X.-X. Wang X. Melnychuk V. Lankford L. Farmer D. (2018). Human amnion extracellular matrix derived bioactive hydrogel for cell delivery and tissue engineering. Mater. Sci. Eng. C 85, 191–202. 10.1016/j.msec.2017.12.026 PMC6353560 29407148

[B174] Sadler T. W. (2022). Langman's medical embryology. Lippincott Williams and Wilkins.

[B175] Salah R. A. Elkhenany H. El-Badri N. (2020). Scaffold engineering using the amniotic membrane. Regen. Med. stem Cell Biol., 323–346. 10.1007/978-3-030-55359-3_11

[B176] Saldin L. T. Cramer M. C. Velankar S. S. White L. J. Badylak S. F. (2017). Extracellular matrix hydrogels from decellularized tissues: structure and function. Acta biomater. 49, 1–15. 10.1016/j.actbio.2016.11.068 27915024 PMC5253110

[B177] Samaniego A. (2011). Allowrap® surgical barrier remains in the body through the cell proliferation phase of the healing process while other surgical barriers are resorbed. AlloSource White Pap.

[B178] Sant'Anna L. B. Hage R. Cardoso M. A. G. Arisawa E. A. Cruz M. M. Parolini O. (2016). Antifibrotic effects of human amniotic membrane transplantation in established biliary fibrosis induced in rats. Cell Transplant. 25 (12), 2245–2257. 10.3727/096368916X692645 27480080

[B179] Sasikumar S. Chameettachal S. Cromer B. Pati F. Kingshott P. (2019). Decellularized extracellular matrix hydrogels—cell behavior as a function of matrix stiffness. Curr. Opin. Biomed. Eng. 10, 123–133. 10.1016/j.cobme.2019.05.002

[B180] Schmiedova I. Ozanova Z. Stastna E. Kiselakova L. Lipovy B. Forostyak S. (2021). Case report: freeze-dried human amniotic membrane allograft for the treatment of chronic wounds: results of a multicentre observational study. Front. Bioeng. Biotechnol. 9, 649446. 10.3389/fbioe.2021.649446 34249879 PMC8264202

[B181] Selmani Z. Naji A. Zidi I. Favier B. Gaiffe E. Obert L. (2008). Human leukocyte antigen-G5 secretion by human mesenchymal stem cells is required to suppress T lymphocyte and natural killer function and to induce CD4+ CD25highFOXP3+ regulatory T cells. Stem cells 26 (1), 212–222. 10.1634/stemcells.2007-0554 17932417

[B182] Serena T. E. Yaakov R. Moore S. Cole W. Coe S. Snyder R. (2020). A randomized controlled clinical trial of a hypothermically stored amniotic membrane for use in diabetic foot ulcers. J. Comp. Eff. Res. 9 (1), 23–34. 10.2217/cer-2019-0142 31691579

[B183] Shao C. Sima J. Zhang S. X. Jin J. Reinach P. Wang Z. (2004). Suppression of corneal neovascularization by PEDF release from human amniotic membranes. Investigative Ophthalmol. Vis. Sci. 45 (6), 1758–1762. 10.1167/iovs.03-0882 15161837

[B184] Shariatzadeh S. Shafiee S. Zafari A. Tayebi T. Yazdanpanah G. Majd A. (2021). Developing a pro-angiogenic placenta derived amniochorionic scaffold with two exposed basement membranes as substrates for cultivating endothelial cells. Sci. Rep. 11 (1), 22508. 10.1038/s41598-021-01922-y 34795361 PMC8602627

[B185] Shimazaki J. Shimmura S. Tsubota K. (2004). Donor source affects the outcome of ocular surface reconstruction in chemical or thermal burns of the cornea11The authors do not have any proprietary interest in the products mentioned used in this study. Ophthalmology 111 (1), 38–44. 10.1016/j.ophtha.2003.02.003 14711712

[B186] Shimmura S. Shimazaki J. Ohashi Y. Tsubota K. (2001). Antiinflammatory effects of amniotic membrane transplantation in ocular surface disorders. Cornea 20 (4), 408–413. 10.1097/00003226-200105000-00015 11333331

[B187] Silini A. R. Cargnoni A. Magatti M. Pianta S. Parolini O. (2015). The long path of human placenta, and its derivatives, in regenerative medicine. Front. Bioeng. Biotechnol. 3, 162. 10.3389/fbioe.2015.00162 26539433 PMC4609884

[B188] Silini A. R. Magatti M. Cargnoni A. Parolini O. (2017). Is immune modulation the mechanism underlying the beneficial effects of amniotic cells and their derivatives in regenerative medicine? Cell Transplant. 26 (4), 531–539. 10.3727/096368916X693699 27938500 PMC5661217

[B189] Slaughter B. V. Khurshid S. S. Fisher O. Z. Khademhosseini A. Peppas N. A. (2009). Hydrogels in regenerative medicine. Adv. Mater. 21 (32‐33), 3307–3329. 10.1002/adma.200802106 20882499 PMC4494665

[B190] Snyder R. J. Shimozaki K. Tallis A. Kerzner M. Reyzelman A. Lintzeris D. (2016). A prospective, randomized, multicenter, controlled evaluation of the use of dehydrated amniotic membrane allograft compared to standard of care for the closure of chronic diabetic foot ulcer. Wounds: a compendium of clinical research and practice. Wounds. 28 (3), 70–77. 26978860

[B191] Srinivasan R. C. Strom S. C. Gramignoli R. (2020). Effects of cryogenic storage on human amnion epithelial cells. Cells 9 (7), 1696. 10.3390/cells9071696 32679793 PMC7407665

[B192] Stern M. (1913). The grafting of preserved amniotic membrane to burned and ulcerated surfaces, substituing skin grafts: a preliminary report. J. Am. Med. Assoc. 60 (13), 973–974. 10.1001/jama.1913.04340130021008

[B193] Swim M. M. Albertario A. Iacobazzi D. Caputo M. Ghorbel M. T. (2019). Amnion-based scaffold with enhanced strength and biocompatibility for *in vivo* vascular repair. Tissue Eng. Part A 25 (7-8), 603–619. 10.1089/ten.tea.2018.0175 30284966

[B194] Tallquist M. D. Molkentin J. D. (2017). Redefining the identity of cardiac fibroblasts. Nat. Rev. Cardiol. 14 (8), 484–491. 10.1038/nrcardio.2017.57 28436487 PMC6329009

[B195] Tan J. L. Chan S. T. Wallace E. M. Lim R. (2014). Human amnion epithelial cells mediate lung repair by directly modulating macrophage recruitment and polarization. Cell Transplant. 23 (3), 319–328. 10.3727/096368912X661409 23294809

[B196] Tehrani F. A. Ahmadiani A. Niknejad H. (2013). The effects of preservation procedures on antibacterial property of amniotic membrane. Cryobiology 67 (3), 293–298. 10.1016/j.cryobiol.2013.08.010 23988559

[B197] Tehrani F. A. Modaresifar K. Azizian S. Niknejad H. (2017). Induction of antimicrobial peptides secretion by IL-1β enhances human amniotic membrane for regenerative medicine. Sci. Rep. 7 (1), 17022–17027. 10.1038/s41598-017-17210-7 29208979 PMC5717175

[B198] Thompson P. Hanson D. S. Langemo D. Anderson J. (2019). Comparing human amniotic allograft and standard wound care when using total contact casting in the treatment of patients with diabetic foot ulcers. Adv. Skin Wound Care 32 (6), 272–277. 10.1097/01.ASW.0000557831.78645.85 31082818

[B199] Toda A. Okabe M. Yoshida T. Nikaido T. (2007). The potential of amniotic membrane/amnion-derived cells for regeneration of various tissues. J. Pharmacol. Sci. 105 (3), 215–228. 10.1254/jphs.CR0070034 17986813

[B200] Tohidi H. Maleki-Jirsaraei N. Simchi A. Mohandes F. Emami Z. Fassina L. (2022). An electroconductive, thermosensitive, and injectable chitosan/pluronic/gold-decorated cellulose nanofiber hydrogel as an efficient carrier for regeneration of cardiac tissue. Materials 15 (15), 5122. 10.3390/ma15155122 35897556 PMC9330822

[B201] Toniato T. V. Stocco T. D. Martins D. S. Santanna L. B. Tim C. R. Marciano F. R. (2020). Hybrid chitosan/amniotic membrane-based hydrogels for articular cartilage tissue engineering application. Int. J. Polym. Mater. Polym. Biomaterials 69 (15), 961–970. 10.1080/00914037.2019.1636249

[B202] Torricelli A. A. Santhanam A. Wu J. Singh V. Wilson S. E. (2016). The corneal fibrosis response to epithelial–stromal injury. Exp. eye Res. 142, 110–118. 10.1016/j.exer.2014.09.012 26675407 PMC4683352

[B203] Tsai G. L. Zilberbrand D. Liao W. J. Horl L. P. (2021). Healing hard-to-heal diabetic foot ulcers: the role of dehydrated amniotic allograft with cross-linked bovine-tendon collagen and glycosaminoglycan matrix. J. Wound Care 30 (7), S47–S53. 10.12968/jowc.2021.30.Sup7.S47 34256586

[B204] Tsai S.-H. Liu Y.-W. Tang W.-C. Zhou Z.-W. Hwang C.-Y. Hwang G.-Y. (2007). Characterization of porcine arterial endothelial cells cultured on amniotic membrane, a potential matrix for vascular tissue engineering. Biochem. biophysical Res. Commun. 357 (4), 984–990. 10.1016/j.bbrc.2007.04.047 17459341

[B205] Výborný K. Vallová J. Kočí Z. Kekulová K. Jiráková K. Jendelová P. (2019). Genipin and EDC crosslinking of extracellular matrix hydrogel derived from human umbilical cord for neural tissue repair. Sci. Rep. 9 (1), 10674. 10.1038/s41598-019-47059-x 31337821 PMC6650505

[B206] Walkden A. (2020). Amniotic membrane transplantation in ophthalmology: an updated perspective. Clin. Ophthalmol. 14, 2057–2072. 10.2147/OPTH.S208008 32801614 PMC7383023

[B207] Wang B. Wang X. Kenneth A. Drena A. Pacheco A. Kalvin L. (2023). Developing small-diameter vascular grafts with human amniotic membrane: long-term evaluation of transplantation outcomes in a small animal model. Biofabrication 15 (2), 025004. 10.1088/1758-5090/acb1da 36626826

[B208] Wassmer C.-H. Berishvili E. (2020). Immunomodulatory properties of amniotic membrane derivatives and their potential in regenerative medicine. Curr. diabetes Rep. 20, 31–10. 10.1007/s11892-020-01316-w PMC7283202 32519069

[B209] Wehmeyer J. L. Natesan S. Christy R. J. (2015). Development of a sterile amniotic membrane tissue graft using supercritical carbon dioxide. Tissue Eng. Part C. Methods 21 (7), 649–659. 10.1089/ten.tec.2014.0304 25471248

[B210] Yadav M. K. Go Y. Y. Kim S. H. Chae S.-W. Song J.-J. (2017). Antimicrobial and antibiofilm effects of human amniotic/chorionic membrane extract on Streptococcus pneumoniae. Front. Microbiol. 8, 1948. 10.3389/fmicb.2017.01948 29089928 PMC5641382

[B211] Yang J. Khan M. Zhang L. Ren X. Guo J. Feng Y. (2015). Antimicrobial surfaces grafted random copolymers with REDV peptide beneficial for endothelialization. J. Mater. Chem. B 3 (39), 7682–7697. 10.1039/C5TB01155H 32264578

[B212] Yazdanpanah G. Jiang Y. Rabiee B. Omidi M. Rosenblatt M. I. Shokuhfar T. (2021). Fabrication, rheological, and compositional characterization of thermoresponsive hydrogel from cornea. Tissue Eng. Part C. Methods 27 (5), 307–321. 10.1089/ten.tec.2021.0011 33813860 PMC8140359

[B213] Yazdanpanah G. Paeini-Vayghan G. Asadi S. Niknejad H. (2015). The effects of cryopreservation on angiogenesis modulation activity of human amniotic membrane. Cryobiology 71 (3), 413–418. 10.1016/j.cryobiol.2015.09.008 26432457

[B214] Yuan Z. Nie H. Wang S. Lee C. H. Li A. Fu S. Y. (2011). Biomaterial selection for tooth regeneration. Tissue Eng. Part B Rev. 17 (5), 373–388. 10.1089/ten.teb.2011.0041 21699433 PMC3179624

[B215] Zare-Bidaki M. Sadrinia S. Erfani S. Afkar E. Ghanbarzade N. (2017). Antimicrobial properties of amniotic and chorionic membranes: a comparative study of two human fetal sacs. J. reproduction Infertil. 18 (2), 218–224. PMCID: PMC5565909. PMC5565909 28868246

[B216] Zasloff M. (2002). Antimicrobial peptides of multicellular organisms. nature 415 (6870), 389–395. 10.1038/415389a 11807545

[B217] Zelen C. M. Gould L. Serena T. E. Carter M. J. Keller J. Li W. W. (2015). A prospective, randomised, controlled, multi‐centre comparative effectiveness study of healing using dehydrated human amnion/chorion membrane allograft, bioengineered skin substitute or standard of care for treatment of chronic lower extremity diabetic ulcers. Int. wound J. 12 (6), 724–732. 10.1111/iwj.12395 25424146 PMC7950807

[B218] Zelen C. M. Serena T. E. Gould L. Le L. Carter M. J. Keller J. (2016). Treatment of chronic diabetic lower extremity ulcers with advanced therapies: a prospective, randomised, controlled, multi‐centre comparative study examining clinical efficacy and cost. Int. wound J. 13 (2), 272–282. 10.1111/iwj.12566 26695998 PMC7949818

[B219] Zhang Q. Chang C. Qian C. Xiao W. Zhu H. Guo J. (2021). Photo-crosslinkable amniotic membrane hydrogel for skin defect healing. Acta Biomater. 125, 197–207. 10.1016/j.actbio.2021.02.043 33676048

[B220] Zhang Y. Fan W. Wang K. Wei H. Zhang R. Wu Y. (2019). Novel preparation of Au nanoparticles loaded Laponite nanoparticles/ECM injectable hydrogel on cardiac differentiation of resident cardiac stem cells to cardiomyocytes. J. Photochem. Photobiol. B Biol. 192, 49–54. 10.1016/j.jphotobiol.2018.12.022 30682654

[B221] Zhou S. Chen J. Feng J. (2003). The effects of amniotic membrane on polymorphonuclear cells. Chin. Med. J. 116 (05), 788–790. 10.3760/cma.j.issn.0366-6999.2003.05.136 12875703

[B222] Zhou Y. Liang K. Zhao S. Zhang C. Li J. Yang H. (2018). Photopolymerized maleilated chitosan/methacrylated silk fibroin micro/nanocomposite hydrogels as potential scaffolds for cartilage tissue engineering. Int. J. Biol. Macromol. 108, 383–390. 10.1016/j.ijbiomac.2017.12.032 29225174

